# Unsupervised ensemble-based phenotyping enhances discoverability of genes related to left-ventricular morphology

**DOI:** 10.1038/s42256-024-00801-1

**Published:** 2024-03-11

**Authors:** Rodrigo Bonazzola, Enzo Ferrante, Nishant Ravikumar, Yan Xia, Bernard Keavney, Sven Plein, Tanveer Syeda-Mahmood, Alejandro F. Frangi

**Affiliations:** 1 Centre for Computational Imaging and Simulation Technologies in Biomedicine, School of Computing and School of Medicine, University of Leeds, Leeds, UK; 2 Leeds Institute of Cardiovascular and Metabolic Medicine, School of Medicine, University of Leeds, Leeds, UK; 3 Research Institute for Signals, Systems and Computational Intelligence, sinc(i), FICH-UNL/CONICET, Santa Fe, Argentina; 4 Division of Cardiovascular Sciences, Faculty of Biology, Medicine and Health, University of Manchester, Manchester, UK; 5 Manchester University NHS Foundation Trust, Manchester Academic Health Science Centre, Manchester, UK; 6 NIHR Manchester Biomedical Research Centre, Manchester Academic Health Science Centre, Manchester, UK; 7 IBM Almaden Research Center, San Jose, CA USA; 8 Division of Informatics, Imaging and Data Sciences, School of Health Sciences, Faculty of Biology, Medicine and Health, University of Manchester, Manchester, UK; 9 Department of Computer Science, School of Engineering, Faculty of Science and Engineering, University of Manchester, Manchester, UK; 10 Medical Imaging Research Center (MIRC), University Hospital Gasthuisberg. Cardiovascular Sciences and Electrical Engineering Departments, KU Leuven, Leuven, Belgium; 11 Alan Turing Institute, London, UK

**Keywords:** Genome-wide association studies, Cardiovascular genetics, Prognostic markers, Machine learning

## Abstract

Recent genome-wide association studies have successfully identified associations between genetic variants and simple cardiac morphological parameters derived from cardiac magnetic resonance images. However, the emergence of large databases, including genetic data linked to cardiac magnetic resonance facilitates the investigation of more nuanced patterns of cardiac shape variability than those studied so far. Here we propose a framework for gene discovery coined unsupervised phenotype ensembles. The unsupervised phenotype ensemble builds a redundant yet highly expressive representation by pooling a set of phenotypes learnt in an unsupervised manner, using deep learning models trained with different hyperparameters. These phenotypes are then analysed via genome-wide association studies, retaining only highly confident and stable associations across the ensemble. We applied our approach to the UK Biobank database to extract geometric features of the left ventricle from image-derived three-dimensional meshes. We demonstrate that our approach greatly improves the discoverability of genes that influence left ventricle shape, identifying 49 loci with study-wide significance and 25 with suggestive significance. We argue that our approach would enable more extensive discovery of gene associations with image-derived phenotypes for other organs or image modalities.

## Abstract (web summary)

Genome-wide association studies allow connecting genomic information with complex traits. Rodrigo Bonazzola et al. develop a framework consisting of several deep learning tools to improve the discoverability of genes that influence specific geometric features of the heart.

## Main

Genome-wide association studies (GWAS) have accelerated the discovery of associations between genomic and complex traits^1^. In general, they analyse genetic variants (that is, the genotype) in a sample of individuals to test their possible association with the presence of disease or with systematic changes in measurable traits, known broadly as phenotypes in this context. GWAS have already successfully identified genetic variants associated with a broad range of diseases and other complex traits, such as metabolic, anthropometric or behavioural ones. These findings have improved our understanding of disease pathogenesis, facilitating the development of better treatments, supporting drug discovery and assisting advances towards precision medicine.

Large-scale epidemiological imaging studies have correlated image-derived phenotypes (IDPs) with genetic data to identify the genetic basis of organ structure and function in health and disease. In cardiology, GWASs have been performed on clinically relevant quantitative left-ventricular (LV) indices, such as LV volumes, LV mass and LV ejection fraction. Diagnosis of patients with heart disease usually involves a quantitative analysis of the LV as a key component^2,3^. Although there are discrepancies in the number of genetic loci associated with changes in LV IDPs from recently reported GWAS^2,4^, some consistent genetic factors have been established.

These cardiac imaging genetics studies were based on traditional approaches, where handcrafted features characterizing LV IDPs were first determined, before running GWAS to find the associated genetic loci. Although these IDPs have been clinically used to diagnose heart disease, they do not provide detailed representations of the chamber morphology and its variation across the population. In this paper, we advance the view that shape features encoded in a learnt latent space can provide a more refined imaging phenotype, which is more informative than traditional measurements. When associated with genetic variation, this can provide novel insights into the genetic basis of cardiac structure and function.

The unprecedented amount of linked genetic and cardiac imaging data available within the UK Biobank (UKBB)^5^ facilitates using unsupervised machine learning techniques to automatically learn a set of characteristics that best describe the morphology of the heart. At the same time, atlas-based methods have been proposed to generate three-dimensional (3D) meshes that represent cardiac anatomy from volumetric images^6,7^. On top of this work, we use the latest advances in graph-convolutional neural networks^8^ to learn low-dimensional representations that consider mesh topology. While standard convolutional neural networks operate on domains with an underlying Euclidean or grid-like structure (for example, images), geometric deep learning generalizes convolutions to non-Euclidean domains such as graphs, meshes and manifolds, taking into account their topology and irregular structure. Previous studies used mesh autoencoders to model the expression space of human face surfaces^9^. Here, we show that such models can enable anatomical variation in cardiac structures to be learnt and correlated with genetic data.

In this work, we learn compact and nonlinear representations of cardiac anatomy in an unsupervised setting via convolutional-mesh autoencoders (CoMA). We propose that the learnt features can identify genetic loci that affect cardiac morphology due to their ability to explain shape variability across the population. We show that such representations can indeed be used to discover novel genetic associations via GWAS, which was not previously possible with traditional handcrafted IDPs such as volume, mass and function indices.

In a previous conference communication^10^, we reported on a much simpler exploratory methodology and analysis, wherein we demonstrated that latent representations learnt from LV surface meshes can find significant genetic associations. In contrast, using latent representations of anatomical meshes of the entire surface, and not just LV functional parameters^2,4,11^ or individual mesh nodes independently^3^ as in previous genetic studies, could reproduce but only marginally expand the knowledge about previously discovered loci. We proposed that this was partially due to the high dimensionality and insufficient expressiveness of the image-derived anatomical phenotypes. In this study, we address these two concerns. First, a new framework, namely, the unsupervised phenotype ensemble (UPE), adds robustness and discoverability: we replicate recently reported genes and discover several novel genetic associations, not yet reported in the literature. Furthermore, this paper expands the size of our cardiac magnetic resonance (CMR) dataset, as well as the accuracy of the derived meshes. We analysed 48,651 participants from the UKBB, deriving high-quality phenotypes and robust latent representations from cardiac segmentations and meshes with a state-of-the-art high-throughput and validated CMR analytic pipeline^12^. We conducted an extensive analysis of the stability of the results. This article underlines the crucial role of high-quality latent representations in imaging genetics to greatly improve gene discoverability associated with LV morphology.

**Fig. 1:**
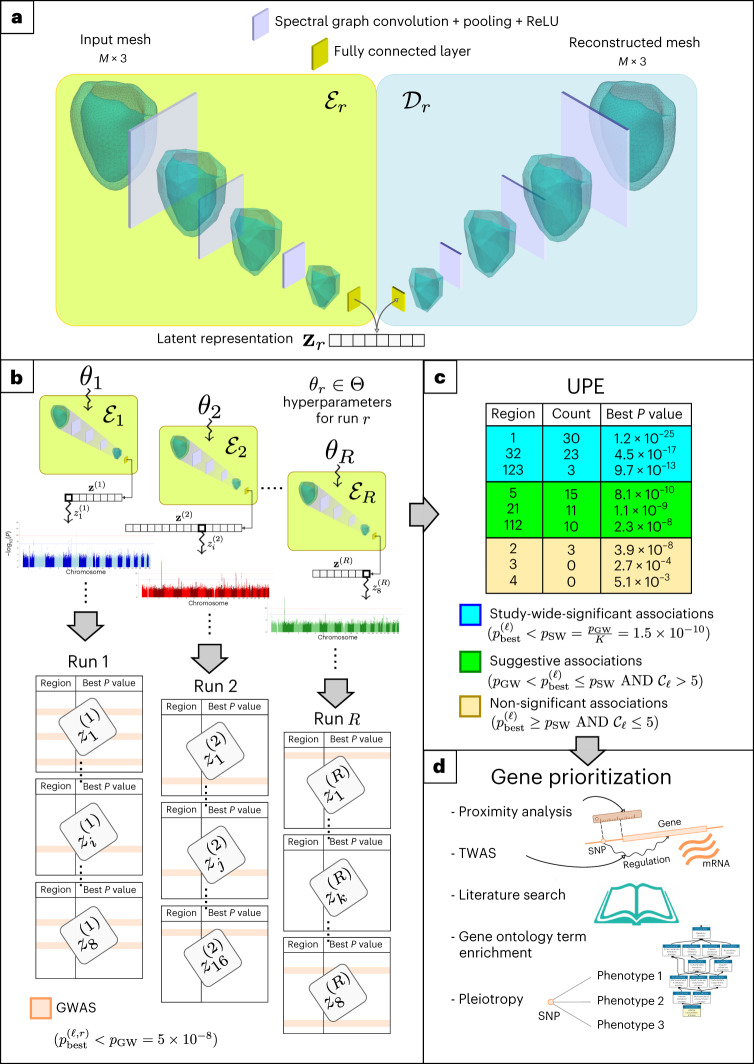
Flowchart of the proposed UPE framework. **a**, A graph-convolutional autoencoder is trained and applied to our set of CMR-derived LV meshes (number of vertices *M* = 5,220) to produce low-dimensional representations of these shapes. In each layer, a representation with fewer vertices is obtained. The bottleneck **z**_*r*_ of the autoencoder with hyperparameters *θ*_*r*_ is a ${n}_{z}^{r}$-dimensional vector for each run *r* (${n}_{z}^{r}\in \{8,16\}$). ReLU, rectified linear unit. **b**, The different latent vectors obtained for each run, ${\{{{{{\bf{z}}}}}_{r}\}}_{r = 1}^{R}$ are then tested in a GWAS component by component, for association with genetic variants. The best association, with *P* value ${P}_{{{{\rm{best}}}}}^{(\ell ,r)}$, is found for each run *r* and region *ℓ* (each region is of around 2 Mb in length). **c**, Associations are then aggregated across the ensemble and classified as significant, suggestive and non-significant according to ${P}_{{{{\rm{best}}}}}^{(\ell )}$ and the count ${{{{\mathcal{C}}}}}_{\ell }$ (as indicated by the rules). **d**, For each significant association, a downstream analysis is conducted to identify potentially causative genes. mRNA, messenger RNA.

A schematic overview of the proposed methodology is presented in Fig. 1. The details of each step are outlined in the Methods section. First, we extracted a surface mesh representation of the anatomical structures. In particular, we studied 3D meshes representing LV at the end of diastole from CMR images of the UKBB database using an automatic deep learning-based segmentation method^12^. We then learn a low-dimensional representation of the 3D meshes, which captures anatomical variations using an encoder–decoder model. All meshes were projected onto this latent space to derive a few shape descriptors (or latent variables) for each of them. GWAS used these features to discover genetic variants associated with shape patterns. Furthermore, to enhance discoverability, we adopt an ensemble-based approach: a set of phenotypes obtained through different models trained and configured with varying network metaparameters and weight initializations (which induce diversity in the learnt representations) are pooled together in one ensemble, yielding redundant yet more expressive representations than the individual latent vectors. The expected improvement of UPE is based on previous work providing evidence that the use of deep ensembles can lead to diverse data representations that are linked in non-trivial ways, even when only the random initialization differs^13^. GWAS is performed against each phenotype of the ensemble, one at a time. A corrected Bonferroni threshold is then calculated to keep the false discovery rate below 5%, by dividing the usual genome-wide threshold by the number of phenotypes of the ensemble being tested.

We demonstrate that this approach effectively discovers additional biologically relevant genetic associations. It expands on previous knowledge by identifying 49 loci with study-wide significance. From this, only nine loci had been reported in previous GWAS of LV phenotypes. This leaves a total of 40 novel LV associations, with eight loci that were reported here in association with handcrafted LV phenotypes, 12 additional associations obtained through shape principal component analysis (PCA) and 20 that are exclusively attributed to our UPE framework with CoMAs. Furthermore, we report 24 suggestive associations, with some highly plausible causative genes according to pre-existing knowledge.

## Results

In the following, we present our GWAS results. First, we investigate handcrafted phenotypes. Second, we examine unsupervised phenotypes obtained via shape PCA. Finally, we examine the results of our proposed UPE approach.

The loci were annotated with gene names on the basis of proximity to the lead single nucleotide polymorphism (SNP) if there was no additional causal evidence in the literature, or with nearby genes likely to mediate the association. For this, we used a diverse array of tools: the functional mapping and annotation (FUMA) web tool^14^, g:Profiler^15^, S-PrediXcan^16^ and the Ensembl Biomart database^17^. Among the candidate genes provided by these tools, a literature review was conducted to find evidence of an association with cardiovascular phenotypes, or experimental. Genes with asterisks were annotated solely on the basis of proximity and hence constitute totally novel findings.

### Genetic findings

#### Handcrafted phenotypes

We performed GWAS on traditional cardiac indices obtained using our segmentation approach. These indices were LVEDV, LV sphericity index at end diastole (LVEDSph), LV myocardial mass (LVM) and LV mass-to-volume ratio (LVMVR = LVM/LVEDV). Note that the LVEDSph as calculated here has not been investigated in previous GWAS (although a related phenotype, named ‘LV internal dimensions’ was studied in an early GWAS of echocardiography-derived LV traits^18^). Details on how to compute this phenotype can be found in the Supplementary Information.

**Extended Data Fig. 1:**
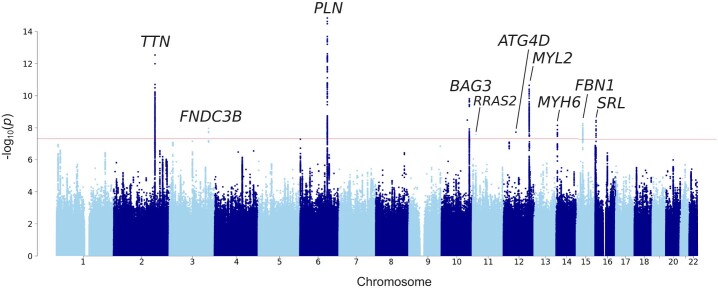
Manhattan plot for GWAS of LVEDV. Manhattan plot of GWAS of left-ventricular end-diastolic volume (LVEDV).

**Extended Data Fig. 2:**
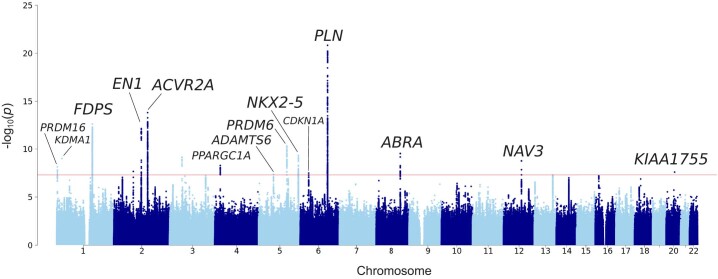
Manhattan plot for GWAS of LVEDSph. Manhattan plot of GWAS of left-ventricular end-diastolic sphericity index (LVEDSph).

**Extended Data Fig. 3:**
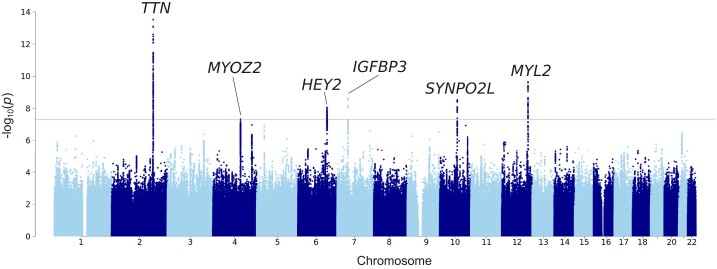
Manhattan plot for GWAS of LVM. Manhattan plot of GWAS of left-ventricular myocardial mass (LVM).

**Extended Data Fig. 4:**
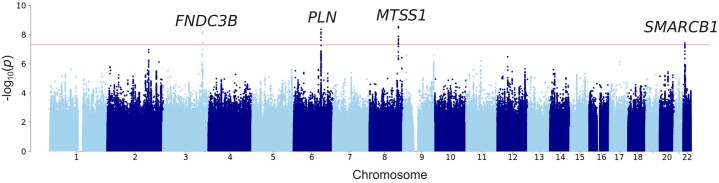
Manhattan plot for GWAS of LVMVR. Manhattan plot of GWAS of left-ventricular mass-to-volume ratio (LVMVR).

In the following, we discuss the associations found for each of these phenotypes. The Manhattan plots are shown in Extended Data Figs. 1–4.

For LVEDV, we discover nine independent associations. The association at intergenic SNP rs11153730 is probably related to *PLN*. This gene plays a crucial role in cardiomyocyte calcium handling by acting as a primary regulator of the SERCA protein (sarco- or endoplasmic reticulum Ca^2+^-ATPase), which transports calcium from the cytosol into the SR1 (ref. ^19^). Mutations in *PLN* have a well-established relationship with dilated cardiomyopathy (DCM)^20^. In ref. ^4^, *PLN* was found to be associated with LVEDV and LVESV. However, ref. ^2^ does not report this locus for the same phenotypes. The locus on chromosome 2 (with lead SNP rs2042995) is widely known to be associated with *TTN*. This gene encodes the protein titin, which is responsible for assembling myocyte sarcomere, and determines the stretching, contraction and passive stiffness of the myocardium^21^. This gene has been reported by refs. ^2,4,11^. rs375034445 lies within the body of *BAG3*; this is a well-known cardiac gene coding for a cellular protein that is predominantly expressed in skeletal and cardiac muscle, which plays a role in myocyte homeostasis and in the development of heart failure^22^; also, it shows a stronger association with LVESV and LV ejection fraction (LVEF), as found in previous studies^2,4^. The locus near the *ATXN2* gene has previously been reported for LVEDV and stroke volume (SV)^4^. A candidate casual gene for this association is gene *MYL2*, the lead SNP (rs35350651) lies 558808 base pairs away from this gene’s transcription start site (TSS)^23^. The gene *TMEM43* has been found in ref. ^4^ in association with LVESV and LVEF. Finally, gene *MYH6* harbours SNP rs365990. This gene provides instructions for making a protein known as the cardiac *α*-myosin heavy chain, which is expressed throughout the myocardium during early cardiac development^24^. Mutations in this gene, as well as the neighbouring *MYH7* responsible for the *β*-myosin heavy chain, have been linked to several pathologies: cardiomyopathies, arrhythmias and congenital heart disease (CHD). Two additional associations are located close to genes *RRAS2* and *ATG4D*, respectively.

For LVEDSph, we find nine additional independent associations, apart from the *PLN* locus. rs35564079 is located 8,250 bp upstream of the TSS of *NKX2-5*, in chromosome 5. This gene plays a crucial role in heart development; in particular, in the formation of the heart tube, which is a structure that will eventually give rise to the heart and great vessels. *NKX2-5* helps determine the heart’s position in the chest and also develops the heart valves and septa. Mutations in the *NKX2-5* gene have been associated with several types of congenital heart defect, including atrial septal defects and atrioventricular block^25^. It has not been reported in refs. ^2^ or ^4^, but shows borderline significance with the fractal dimension of the LV trabeculae^11^. rs72007904 is located 300 kb upstream of the TSS of the gene *ABRA*. *ABRA* codes for a cardiac and skeletal muscle-specific actin-binding protein located in the Z disc and M-line and binds with actin. Consistent with this, it is differentially expressed in cardiac tissues and skeletal muscle in the genotype-tissue expression (GTEx) data. *ABRA* has been associated with DCM in mice^26^. rs35001652 is close to *KDM1A*, a gene that codes for a histone demethylase involved in cardiac development, according to studies in mice^27^. rs463106 lies in the body of gene *PRDM6*. The mouse homologue of this gene, *Prdm6*, has been found to be important in early cardiac development^28^. An interesting association, with SNP rs162746, is close to gene *EN1*, however, we were not able to find a strong candidate gene in this region. Finally, rs573709385 lies in a gene desert in chromosome 2, the closest protein-coding genes are *ACVR2A* and *ZEB2* (both at around 1.6 Mb).

For LVM, four associations are found: rs4767239 is probably related to developmental gene *TBX5* (T-box transcription factor 5), which has a known role in developing the heart and the limbs^29^. Through familial studies, mutations in this gene have been associated with Holt-Oram syndrome, a developmental disorder affecting the heart and upper limbs. In particular, there have been no recent reports on GWAS on LV phenotypes. The locus near the *CENPW* gene has a cardiac gene, *HEY2*, possibly causal for this association. *HEY2* has been shown to suppress cardiac hypertrophy through an inhibitory interaction with *GATA4*, a transcription factor that plays a key role in cardiac development and hypertrophy^30^. *HEY* proteins are direct targets of Notch signalling and have been shown to regulate multiple key steps in cardiovascular development. Studies have found that the loss of *HEY2* in mice leads to cardiac defects with high postnatal lethality^31^. This locus has also been reported as associated to right-ventricular phenotypes^32^. rs3740293 overlaps gene *SYNPO2L*, which is highly expressed in cardiac tissues (LV and atrial appendage) and skeletal muscle, making it a strong candidate gene. This SNP is also close to gene *MYOZ1*, which is also supported by our GWAS study (section on transcriptome-wide association studies, ‘TWAS’). Both genes have been previously proposed as candidates for cardiac phenotypes, in particular atrial fibrillation^33,34^. However, *MYOZ1* shows very high expression only in the latter. Loss-of-function variants in this *SYNPO2L* have also been found causative of atrial fibrillation^35^, supporting this gene as a more likely candidate. rs73243622 is close to the candidate gene *PPARGC1A*. Finally, gene *CDKN1A* has been found in ref. ^4^ in association with LVESV and LVEF. Finally, for LVMVR, three new loci were found, apart from the *PLN* locus: rs2070458 close to *SMARCB1* (in chromosome 22), rs17460016 in the *FNDC3B* locus (in chromosome 3) and rs12542527 (in chromosome 8). The last is an eQTL for the *MTSS1* gene also linked to LV fractal dimension^11^.

The detailed summary statistics for the significant associations with handcrafted phenotypes are provided as Supplementary Data.

#### Shape PCA

A shape PCA model was fit to our set of meshes (Methods). The effect on LV shape for the first 16 modes is shown in the Supplementary Fig. 7. GWAS was performed for these 16 modes and 18 independent loci were found with study-wide significance (*P* < 3.1 × 10^−9^). PC1, which is highly correlated with LVEDV, reconfirms the associations with *TTN*, *MYL2* and *MYH6*. A new association, in chromosome 4, is an indel (chr4:120304290_GC_G) located 200 kb downstream of *MYOZ2*. This gene codes for protein that functions by tethering calcineurin to alpha-actinin at Z-discs in muscle cells and inhibits the pathological cardiac hypertrophic response^36^. Another candidate gene in this locus is *PDE5A*. Indeed, some of the strongest associations overlap the body of this gene (although not the lead variant, which is the indel mentioned above). It has been shown that *PDE5A* is expressed in cardiac myocytes and may have pro-hypertrophic effects^37^.

PC2 is strongly linked with a new locus in chromosome 17, *GOSR2*. This component seems to be linked to LV conicity. Ref. ^11^ reports the *GOSR2* locus as significantly associated with trabecular fractal dimension in slices 3 and 4, however, previous GWAS in global LV indices have not reported this locus. More broadly in the literature on genetics of cardiovascular phenotypes, it has been reported as associated to ascending aorta distensibility^38^, mitral valve geometry^39^ and CHD^40^.

PC3, highly correlated with LVEDSph, re-discovers the *PLN* and *NKX2-5* loci. It also adds an association in chromosome 1, the SNP rs12142143, which lies within the *ACTN2* gene. This gene codes for the Z disc protein *α*-actinin-2. This locus has been reported for SV in ref. ^4^.

PC6 has hits in the *TBX5* and *NKX2-5* loci, with a new association near the *NAV3* gene, that has been found to play a role in heart development in zebrafish^41^. PC7 is associated to a SNP near the TSS of *PITX2* gene. It encodes for a transcription factor required for mammalian development, and disruption in its expression in humans causes CHD and is associated with atrial fibrillation. PC10 is linked to the *PRDM6* locus (discussed before in connection with LVEDSph). PC11 is associated to SNPs rs59894072 (close to *TBX3*, a known cardiac gene^42^) and rs56229089. The second, in turn, is close (1 Mb) to two possible candidate genes: *KCNJ2*, a potassium channel gene that is active in skeletal muscles and cardiac muscles^43^ and *SOX9*, a gene implicated in cardiac development^44^. The detailed summary statistics for the significant associations with shape PCs are provided in Supplementary Data.

#### UPE

CoMAs were trained on LV meshes at end diastole, using a range of network hyperparameters. The reconstruction performance for these models is shown in Supplementary Fig. 1.

GWAS was performed on all latent variables, for all training runs achieving a good reconstruction performance (Methods). A run is an instance of model training, defined by the choice of hyperparameters: in particular, random seeds controlling training and validation samples, weight initialization, network architecture and Kullback–Leibler divergence weight. The number of such runs was *R* = 36. The results obtained with *n*_*z*_ = 8 and *n*_*z*_ = 16 (8 and 16 latent variables, respectively) are reported, with a total number of 384 latent variables in the pooled representation. First, we examine the prevalence of significant GWAS loci found in all runs of our ensemble. To count the loci, we split the genome into approximately linkage disequilibrium-independent genomic regions^45^ and computed the number of loci below the usual genome-wide significance threshold of 5 × 10^−8^ (see details in the Methods section); Table 1 shows the results.

**Table 1:** Counts of GWAS hits across runs in the UPE framework, ${{{{\mathcal{C}}}}}_{\ell }$ for each locus *ℓ*, which represents the number of runs for which the corresponding locus shows at least one association with *P* < *P*_GW_ = 5 × 10^−8^ (see details in the Methods section)

Chromosome	Region	Candidate gene	Count	Minimum *P* value	Lead variant	NEA/EA	EAF (%)	$\bf{|} \hat{\boldsymbol{\beta} }| \pm {{{\bf{se}}}}(\hat{\boldsymbol{\beta} })({\boldsymbol{\times 1{0}^{-2}}})$
10	120591353–122407323	BAG3	35	4.1 × 10^−18^	rs375034445	A/AT	21.2	5.29 ± 0.79
2	178553183–181312739	TTN	35	1.4 × 10^−17^	rs2042995	T/C	23.2	5.70 ± 0.77
6	117672972–118963115	PLN	35	2.0 × 10^−29^	rs11153730	T/C	48.6	4.29 ± 0.64
14	23018665–24905123	MYH6	34	2.7 × 10^−14^	rs365990	A/G	36.9	4.04 ± 0.66
12	113986709–115036602	TBX5	34	2.3 × 10^−11^	rs4767239	G/C	82.5	4.12 ± 0.85
12	110336719–113263518	MYL2	34	2.8 × 10^−15^	rs35350651	A/AC	51.4	4.36 ± 0.64
4	119933512–120392684	MYOZ2	33	2.4 × 10^−13^	4:120304290_GC_G	GC/G	29.0	3.96 ± 0.71
10	73508512–75422550	SYNPO2L	31	2.5 × 10^−15^	rs3740293	A/C	14.3	5.07 ± 0.92
3	157312028–159477890	**SHOX2**	30	7.0 × 10^−15^	rs11706187	A/G	50.1	3.23 ± 0.64
1	154770403–156336133	**FDPS**	29	9.7 × 10^−13^	rs41314549	T/C	2.81	13.7 ± 1.9
17	43056905–45876022	GOSR2	26	8.3 × 10^−22^	rs17608766	T/C	14.3	5.73 ± 0.90
3	99373762–100592217	**FILIP1L**^a^	25	8.4 × 10^−14^	rs9811920	G/A	40.8	3.23 ± 0.65
16	4001196–5118345	**SRL**	24	9.4 × 10^−12^	rs889807	T/C	50.8	3.24 ± 0.64
1	21736588–23086883	KDM1A	22	2.0 × 10^−12^	rs35001652	G/A	37.3	2.59 ± 0.67
7	45952922–46986720	IGFBP3	22	2.0 × 10^−11^	rs143741275	A/AGTGTGT	42.4	2.59 ± 0.66
5	171074292–172678327	NKX2-5	21	9.0 × 10^−14^	rs35564079	C/CT	28.5	2.76 ± 0.72
3	13070799–14816900	TMEM43	21	2.7 × 10^−11^	rs900173	T/C	34.0	3.48 ± 0.68
1	144977494–148361253	**GJA5**	20	1.1 × 10^−10^	rs12046416	A/G	33.5	2.81 ± 0.68
1	235819436–237555628	ACTN2	20	2.0 × 10^−12^	rs12142143	T/C	53.1	4.11 ± 0.65
13	20686720–22242174	FGF9	20	8.8 × 10^−13^	rs10628955	G/GAA	47.7	3.95 ± 0.67
4	111256567–113870102	PITX2	19	4.4 × 10^−14^	rs2723294	C/T	69.5	4.65 ± 0.70
1	14891511–16897730	HSPB7	17	2.3 × 10^−11^	rs1763605	T/G	67.5	2.80 ± 0.68
2	146445570–147277162	ACVR2A/ZEB2	16	2.9 × 10^−11^	rs573709385	A/AT	44.9	1.93 ± 0.65
6	125424383–127540461	HEY2	16	2.9 × 10^−11^	rs11423823	C/CT	50.2	3.23 ± 0.66
2	36122006–38132712	**STRN**	15	9.9 × 10^−16^	rs2110944	T/C	52.6	3.71 ± 0.64
16	79134815–80297374	**MAF**^a^	15	5.2 × 10^−11^	rs558328129	A/AT	45.4	2.97 ± 0.70
8	107410754–108648177	ABRA	15	8.1 × 10^−11^	rs72007904	A/AACTATTC	50.0	3.87 ± 0.64
21	27271019–29125226	**ADAMTS1**	15	1.4 × 10^−10^	rs2830977	G/A	22.0	4.29 ± 0.78
1	49894177–51713726	**RNF11**^a^	13	3.1 × 10^−11^	rs7555411	C/T	1.42	13.8 ± 2.7
13	98938919–100574095	**DOCK9**^a^	13	1.2 × 10^−10^	rs34138434	C/A	29.7	4.62 ± 0.72
2	118367466–121303783	EN1^a^	13	6.7 × 10^−14^	rs162746	A/G	67.5	4.01 ± 0.68
12	27799773–29651255	**CCDC91**^a^	12	2.0 × 10^−14^	rs5797270	G/GT	20.3	3.35 ± 0.81
13	25784362–27284362	WASF3^a^	11	9.3 × 10^−11^	rs61944841	G/A	41.3	3.07 ± 0.67
18	19485844–20649472	**GATA6**	11	6.6 × 10^−11^	rs62094198	T/A	39.6	2.21 ± 0.66
14	19002084–21589402	**NDRG2**	10	2.5 × 10^−11^	rs12889267	A/G	16.7	3.43 ± 0.85
6	35455756–37572596	CDKN1A	10	3.2 × 10^−13^	rs3176326	G/A	19.8	3.47 ± 0.81
7	118351581–121045273	**WNT16**	10	2.4 × 10^−11^	rs3801387	A/G	28.1	2.79 ± 0.72
16	75977954–77523678	**ADAMTS18**	10	5.2 × 10^−13^	rs62046468	C/T	37.6	4.80 ± 0.66
17	41772087–43056905	**SOST**	10	5.5 × 10^−11^	rs17881550	G/GC	43.4	3.65 ± 0.65
11	27020461–28481593	**CCDC34**^a^	8	5.7 × 10^−12^	rs10835164	C/T	25.6	3.84 ± 0.74
5	120452166–122556905	PRDM6	8	6.2 × 10^−11^	rs463106	T/C	47.2	3.68 ± 0.65
17	67858770–69387817	KCNJ2/SOX9	7	3.6 × 10^−12^	rs56229089	G/C	55.7	2.89 ± 0.65
5	63968304–65911286	ADAMTS6	7	1.6 × 10^−11^	rs753963943	ATT/A	42.5	2.88 ± 0.66
18	8498931–11075913	**NDUFV2**	6	6.1 × 10^−12^	rs206524	T/C	70.7	3.01 ± 0.71
11	65898631–68005825	**KDM2A**	4	2.1 × 10^−11^	rs12785906	G/C	5.84	7.40 ± 1.38
1	1892607–3582736	PRDM16	3	1.7 × 10^−11^	rs781212641	G/GC	9.33	5.68 ± 1.12
4	7539692–8152235	**AFAP1**	3	1.4 × 10^−11^	rs28542374	G/A	63.5	3.10 ± 0.67
12	76511314–78570570	NAV3	3	2.7 × 10^−12^	rs7965680	T/C	55.7	2.43 ± 0.65
17	15019097–16412342	**CENPV**^a^	3	3.4 × 10^−11^	rs7477	A/C	49.8	3.18 ± 0.64

The total number of runs was 36. The lead variant is the one for which the minimum *P* value occurs. *P* values are two-sided and derived from a linear association *t*-statistic (no adjustments were made for multiple comparisons). NEA and EA stand for non-effect and effect allele, respectively, whereas $\hat{\beta }$ is the standardized effect size estimate. EAF is the frequency of EA. Note that these values correspond to different phenotypes of the ensemble, therefore they are not comparable in terms of the magnitude of the morphological change that they produce. The directions of effect can be understood with the help of Supplementary Table 5.

^a^The genes were annotated based purely on closest proximity to the lead variant in that region (the rest of the genes have additional previous evidence of a link to cardiac physiology as discussed in the text).

Gene names that are in bold mean that the corresponding locus is only discovered with UPE, that is the remaining loci were already found by testing the handcrafted phenotypes or the shape PCs.

We found 49 independent associations with study-wide significance. All of the previously discussed findings are recovered by UPE with study-wide significance, except the following loci: *MTSS1*, *TBX3*, *PPARGC1A* and *FNDC3B* (the last two show with suggestive significance in UPE). The summary statistics of the GWAS for the best latent variable of each of these 49 loci are displayed in Table 1. When a gene name is displayed in bold letters, it means that this locus was found only via the ensemble approach. Most loci have previous evidence supporting their plausible role in cardiac pathways. In addition, many of them are totally novel and represent interesting avenues for further research.

In what follows, we perform an in-depth analysis of our novel genetic findings in the light of recent literature.

##### Loci with previous evidence

We now describe loci that have not been linked to structural LV phenotypes in recent GWAS, but count with other types of evidence.

rs11706187 is probably linked to developmental gene *SHOX2*. The mouse homologue of *SHOX2*, *Shox2*, is essential to differentiate cardiac pacemaker cells by repressing *Nkx2-5* (ref. ^46^). Whereas both *TBX5* and *NKX2-5* are highly expressed in adult cardiac tissues according to GTEx data, *SHOX2* is not highly expressed in these tissues. A possible hypothesis is that rs11706187 regulates the expression of *SHOX2* in developmental or pre-adult stages.

A particularly interesting association, with the SNP rs2245109, is located within the body of the *STRN* gene on chromosome 2 and is probably causally related to it: this gene encodes the protein striatin, which is expressed in cardiomyocytes and has been shown to interact with other proteins involved in the mechanism of myocardial function^47^. Mutations in this gene have been shown to lead to DCM in dogs^48^. In humans, there has been a recent GWAS on heart failure that reported this locus, but our study links it with cardiac morphology. Moreover, our estimated effect size is substantially higher; suggesting that this latent variable is an endophenotype closer to the underlying biology. This could provide insight to unravel the aetiology of a heterogeneous condition such as heart failure. The lead SNP has a high minor allele frequency (MAF) of 47.4%. This locus also contains eQTLs for this gene, as evidenced by TWAS (section ‘TWAS’). Something similar occurs with the *RNF11* locus, although this does not reach genome-wide significance for heart failure (*P* = 3.2 × 10^−6^). The lead variant for this locus is an indel with low frequency (MAF 1.4%) and large estimated standardized effect size ($\hat{\beta }=$ 0.138). This locus has also been linked to the QRS (a combination of the Q, R and S waves) interval, although the causative gene is not clear^49^, some candidates being *RNF11* itself, *CDKN2C*, *C1orf185* and *FAF1*.

The *SRL* gene, which encodes the sarcalumenin protein, harbours the SNP rs889807. Sarcalumenin is a protein that binds Ca^2+^ located in the longitudinal sarcoplasmic reticulum of the heart. Its main function is to regulate Ca^2+^ reuptake in the sarcoplasmic reticulum by interacting with the cardiac sarco (endo)plasmic reticulum Ca^2+^-ATPase 2a (SERCA2a). According to GTEx data, this gene is highly expressed in adult cardiac tissue (both in the LV and atrial appendage tissues) and skeletal muscle.

Several associations lie near genes of the *ADATMS* (a disintegrin and metalloproteinase with thrombospondin motifs) family^50^: *ADAMTS1* and *ADAMTS5* (near rs2830977 on chromosome 21, with *P* = 1.4 × 10^−10^), *ADAMTS6* (rs753963943 on chromosome 5, *P* = 5.6 × 10^−11^) and *ADAMTS18* (chromosome 16, *P* = 5.2 × 10^−13^).

An association lies 260 kb upstream of *GATA6*, a transcription factor that plays a critical role in the development of the heart. It has been found to regulate the hypertrophic response^51^. Sequence variants in this gene have been discovered to predispose for CHD phenotypes^52,53^.

rs12889267 lies 3,700 kb upstream of the TSS of *NDRG2*. This gene has been demonstrated to play a role in protection against ischaemia and/or reperfusion injury, in a study in rats^54^.

One SNP overlaps *KDM2A*. As *KDM1A*, it is a histone demethylase gene. Although its link to the heart is less clear, there exists evidence from knockout studies in mice that supports its importance in embrionic development, including heart development^55^.

rs206524 is located within a gene for long non-coding RNA, *LINC01254*. A possible candidate protein-coding gene is *NDUVF2*, located 1.3 Mb upstream of the SNP. According to the GTEx dataset, *NDUFV2* is highly expressed in cardiac and skeletal muscle tissue.

rs12046416 is located 8,268 bp upstream of the TSS of *GJA5*, a gene that is expressed in atrial myocytes and mediates the coordinated electrical activation of the atria^56^.

##### Novel loci

In addition to the loci with previous evidence discussed above, we report a number of novel genetic loci with *P* < *P*_SW_, which have not been previously reported in connection with cardiac phenotypes or pathways. These loci were annotated on the basis of the closest gene: *CCDC91*, *FILIP1L*, *EN1*, *AFAP1*, *IGFBP3*, *CCDC34*, *WASF3*, *DOCK9* and *MAF*. Of particular interest are those loci with a small number of counts, for example ${{{{\mathcal{C}}}}}_{\ell }\le 15$. These are the loci for which the ensemble approach seems most relevant, since they are unlikely to be pinpointed by one particular run. Furthermore, they are typically not found by testing the shape PCs, as evidenced by the higher frequency of bold letters towards the bottom of Table 1.

##### Loci with suggestive significance

In addition to genetic loci with *P* < *P*_SW_, several SNPs show *P*_SW_ < *P* < *P*_GW_ in five or more independent runs. We consider these associations suggestive and briefly discuss some of them here. The summary statistics for these associations are shown in Supplementary Table 3. Some of these loci have been found in previous studies: GWAS studies, familial studies or studies with model organisms. For example, variants in gene *RBM20* are associated to DCM^57^. We observe that the lead SNP in this region has a low MAF (1.4%), and the effect size estimate is high (standardized $\hat{\beta }=$ 0.20).

A cluster of associations in chromosome 1 is located in a region that includes the *S100* family of genes. In particular, the lead SNP in this region, rs985242, is located within the genes *S100A1* and *S100A13*. The S100 is a family of low-weight Ca^2+^-binding EF-hand proteins, with 25 human genes identified.

The SNP rs28681517 lies within gene *ADAMTSL3*, whose associated protein has been shown to play a crucial role in maintaining cardiac structure and function in mice^58^.

SNP rs569550 lies 578,846 base pairs away from *KCNQ1*, which belongs to a large family of genes that provide instructions for making potassium channels. *KCNQ1* encodes the alpha subunit of the potassium channel KvLQT1. Mutations in *KCNQ1* are responsible for the long QT syndrome^59^.

Deletion 15:48690566_TC_T is a relatively common variant (MAF 14.4%), and is located 10 kb downstream of the transcription end site of *FBN1*. Mutations in this gene are associated with Marfan syndrome, a genetic disorder that affects connective tissues in the body. It can have various manifestations, including cardiovascular complications.

rs9814240 is a coding variant in the *LMCD1* gene. Mutations in this gene are causative of hypertrophic cardiomyopathy in mice^60^, however, no association had been found between variants in this gene and human cardiac phenotypes. Moreover, this gene has been found to interact with (the homologous of) *GATA6* in mice^61^. *GATA6* is located near one of the loci discovered with study-wide significance.

### Effect on LV morphology

**Fig. 2:**
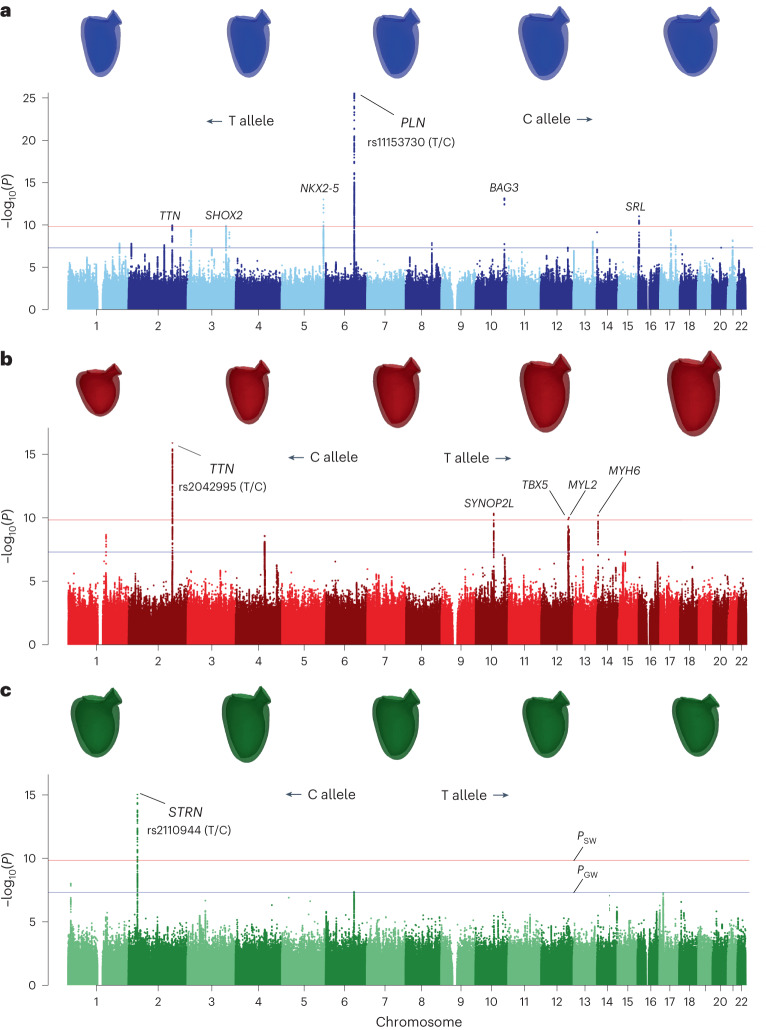
Variants in the *TTN*, *PLN* and *STRN* loci show distinct effects on LV morphology. **a**–**c**, Manhattan plots for LV latent variables with best association for SNPs at the *PLN* (**a**), *TTN* (**b**) and *STRN* (**c**) loci. On top are shown the average meshes corresponding to the following range of quantiles, for each latent variable (from left to right): [0, 0.01], [0.095, 0.105], [0.495, 0.505], [0.895, 0.905] and [0.99, 1].

The effect of these loci on the LV morphology was evaluated by selecting the single phenotype with the strongest *P* value for the associated locus. To help characterize these latent variables, the Spearman correlation coefficient between the latter and the handcrafted LV indices were calculated and shown in Supplementary Table 4. We also examine the shapes of the average mesh within different ranges of quantiles for this latent variable, from 0 through 1. This is shown in Fig. 2, along with the associated Manhattan plots, for the loci *PLN*, *TTN* and *STRN*. The direction of effect is shown by indicating with arrows which allele favours which shape. We observe a very distinct effect on the morphology of each of these SNPs. While the *PLN* variant influences a latent variable that has a a smaller effect on LVEDV (Spearman *r* = 0.722) and a strong link to LVEDSph (*r* = 0.532), the best latent variable for *TTN* gene shows a greater correlation with LVEDV (*r* = 0.910). Consistent with this, the GWAS on LVEDSph shows no significant signal for *TTN*, but a strong one for *PLN* (*P* = 10^−20^, Extended Data Fig. 2), which is also in line with a previous finding of ours^10^. Furthermore, these findings are in line with the effects of PC1 and PC3, where *TTN* and *PLN* loci are found, respectively.

The SNP in the *STRN* gene is associated with a subtle phenotype that controls mitral orientation without a concomitant change in LV size (Fig. 2). This is consistent with the fact that it was not discovered in previous studies of structural LV phenotypes. Notably, this effect is consistent with the observed effect of PC4, for which this locus reaches genome-wide significance (see Supplementary Fig. 2 for the effect of PC4).

### TWAS

We performed TWAS using the S-PrediXcan tool^16^, to test the possibility of a mediating effect of gene expression and intron excision events on structural phenotypes. This tool is fed with models that impute gene expression and intron excision data on the basis of the genotype, which in turn were trained using data from the GTEx project, v.8 (ref. ^62^).

Our focus was on cardiovascular tissues, specifically the LV, atrial appendage and coronary, aortic and tibial arteries. To maintain statistical rigour, we applied a significance threshold of *P*_GE*x*_ = 2.2 × 10^−9^, which adjusts for multiple comparisons (324 phenotypes and 68,919 tissue–gene pairs). Similarly, for alternative splicing, the threshold was set at *P*_AS_ = 8.2 × 10^−10^, considering the same multiple testing correction (187,535 being the number of intron-tissue pairs tested).

In the cardiac tissues (LV and atrial appendage), we identified genes located within loci of previously reported genes. In the LV, these included *NKX2-5*, *STRN*, *SYNPO2L* (*FUT11*, *SEC24C* and *SYNPO2L* itself), *PLN*, *HEY2* (*CENPW* gene), *TTN* (*FKBP7* gene), *CENPV*, *GOSR2* (*MAPT* and *GOSR2* itself) and *FDPS* (*SCAMP3*, *ARHGEF2*, *RIT1*, *GOSR2*, *MAPT*, *HCN3*, *GBA*, *MSTO1*, *RUSC1*, *FUT11*, *SYT11*, *ADAM15* and *FDPS* itself). For the atrial appendage, the genes included *PLN*, *STRN*, *NKX2-5*, *SYNPO2L* and *MYOZ1* within the *SYNPO2L* locus, as well as *FKBP7* and *SCAMP3*. Many of these genes had been previously implicated on the basis of independent knowledge, bolstering the evidence for their potential causal roles. Notably, our analysis also revealed the direction of the effect on gene expression: higher *PLN* expression was associated with a more spherical LV morphology, while lower *NKX2-5* expression was linked to the same phenotype (refer to Fig. 2b). Furthermore, an elevated *STRN* expression (in both cardiac tissues) was associated with a more horizontal mitral orientation (Fig. 2c). Detailed results for significant gene expression associations are provided as Supplementary Data.

In the case of arterial tissues, we found significant associations within various loci, such as the *SYNPO2L* locus (with the genes *AGAP5*, *FUT11*, *SEC24C* and *ARHGAP27*), *FDPS* (*ARHGEF2*, *CLK2*, *FAM189B*, *GBA*, *GON4L*, *HCN3*, *NPR1* and *SYT11*), *CENPW*, *TTN* (*PRKRA* and *FKBP7* genes), *PLN* (*CEP85L* and *PLN*), *GOSR2* (*WNT3*, *CRHR1*, *LRRC37A* and *MAPT*), *KDM2A*, *LINC01562*, *MYH6* (*MYH6* and *MYH7*), *RP11-383I23.2*, *RP11-574K11.29*, *SCAMP3*, *MYL2* (*SH2B3* gene), *SOST* and *TCF21*.

Detailed results for intron excision events are provided in Supplementary Data.

### Gene ontology enrichment analysis

We use the tool g:Profiler to find pathways for which our sets of genes were enriched. To define the gene sets, we selected a region of 100 kb around each lead variant and chose the genes whose TSS was located within that window. Gene ontology terms belong to one of three different categories: molecular functions, cellular components and biological processes. Within the cellular component category, we have found a relevant enriched term, ‘Sarcomere’, comprising the following nine genes from our query: *ACTN2*, *MYOZ1*, *SYNPO2L*, *BAG3*, *TNNT3*, *TNNI2*, *MYH6*, *MYH7*, *KY* (*P* = 9.2 × 10^−3^). Within the biological process category, the terms ‘Myofibril assembly’, ‘striated muscle cell development’ and ‘sarcomere organization’ result enriched (*P* = 1.2 × 10^−3^, *P* = 1.4 × 10^−3^ and *P* = 1.5 × 10^−3^, respectively). Within the molecular function category, the term ‘calcium-dependent protein binding’ is enriched (*P* = 2.9 × 10^−8^), although it is composed of nine members of the S100A family (which encompass a single locus), apart from *SYT8* and *TNNT3*.

### Phenome-wide association studies

To detect pleiotropic effects, we performed a phenome-wide association study of the lead SNPs from Table 1. For this, we queried the Integrative Epidemiology Unit OpenGWAS Project’s database. The results are included in the Supplementary Data File. We discuss briefly here some associations with cardiovascular phenotypes. A number of loci were associated to cardiac electrical phenotypes: *CDKN1A*, *NDRG2*, *PLN*, *TBX5* and *MYH6*. The following loci were associated to pulse rate: *SYNPO2L*, *NDRG2*, *MYH6*, *SRL*, *GOSR2*, *GATA6*, *ACTN2*, *KIAA1755*, *TMEM43*, *SLC27A6* and *FNDC3B*. The lead SNP at the *PRDM6* locus was associated to heart rate recovery post exercise. The following loci were associated to blood pressure phenotypes (diastolic, systolic or hypertension): *SYNPO2L*, *KCNQ1*, *MYL2*, *NDRG2*, *MYH6*, *SRL*, *GOSR2*, *GATA6*, *HSPB7*, *RNF11*, *EFEMP1*, *FNDC3B*, *NME9*, *PRDM6* and *PLN*. Finally, *SYNPO2L*, *TBX5*, *MYH6*, *GOSR2*, *PITX2* and *CDKN1A* were associated to cardiac arrhytmias.

### Replication study

We set apart a subset of 5,470 UKBB participants of British ancestry for which the whole pipeline was run identically to the individuals from the discovery set. We report the detailed results in the Supplementary Material, including the estimated statistical power for each SNP on the basis of the effect size estimate $\hat{\beta }$ from the discovery phase. Among the 49 study-wide significant loci, we report 28 that replicate with *P* < 0.05 (whereas seven replicate with the more stringent Bonferroni threshold of *P* < 0.05/49), as well as 47 loci for which the estimated direction of effect is consistent with that found in the discovery phase. For the suggestive associations, 11 loci replicated (out of 25) with the threshold of *P* < 0.05, whereas 22 have a concordant direction of effect between the discovery and replication phases.

### Comparison with GWAS on traditional LV indices

For comparison, we collected the GWAS summary statistics from previous studies on LV phenotypes, derived also from UKBB CMR images, namely refs. ^2,4^ and ^11^. We also include the results for LVESV, SV and LVEF from these studies. However, note that the unsupervised features studied in this work are static and were extracted using only the end-diastolic phase.

**Fig. 3:**
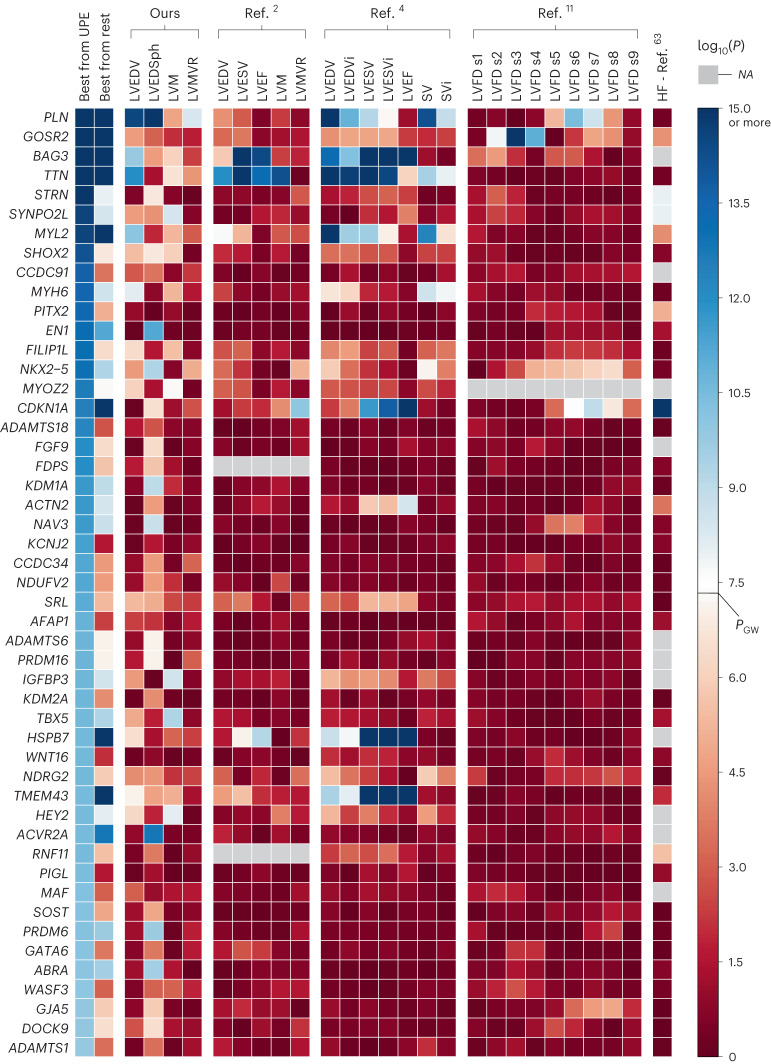
Comparison of the $\small{-{\mathbf{log} }_{\mathbf{10}}({\mathbf{P}})}$ values for the lead variants of the 49 study-wide significant genetic loci found in this work, with GWAS on handcrafted cardiac indices and a GWAS on heart failure. The leftmost column corresponds to the best association found for that locus across the ensemble of phenotypes, whereas the second column corresponds to the best *P* value for that locus across the previous GWAS, where the *P* values are two-sided and derived from a linear association *t*-statistic (no adjustments were made for multiple comparisons). The white colour corresponds to the genome-wide significance threshold of 5 × 10^−8^, whereas the shades of red and blue correspond to weaker and stronger associations, respectively. SV denotes stroke volume. LVEDVi, LVESVi and SVi denote the indexed versions of the phenotypes, that is, the phenotype divided by the participant’s body surface area. Finally, LVFD s*n* stands for LV trabecular fractal dimension measured at the *n*th slice of the LV longitudinal axis (for details, refer to the original publication in ref. ^11^). Grey squares (NA, not applicable) mean that the genetic variant was not tested in the corresponding study. Source data

The comparison can be seen in Fig. 3. For each locus in Table 1 (which all pass the Bonferroni threshold), this figure displays the association *P* value found in previous GWAS and on our own GWAS of handcrafted phenotypes. Shades of red represent non-genome-wide significant associations, whereas shades of blue represent genome-wide significant ones and white corresponds to the *P*_GW_ threshold. The second column represents the best *P* value across all traditional phenotypes for the loci given in the columns. Therefore, a shade of red in this column means that the locus is novel in the context of LV structural phenotypes.

## Discussion

As shown in ‘Results’, we were able to retrieve study-wide significant loci that had been found in previous GWAS on handcrafted phenotypes (*PLN*, *TTN*, *MYL2*, *GOSR2*, *BAG3*, *TMEM43*, *HSPB7*, *CPKN1A*, *NKX2-5*). Furthermore, genes with a known role in cardiac physiology (for example, *TBX5*, *SHOX2* and *STRN*) were identified, but no previous association with GWAS of LV phenotypes had been found in previous studies. Thirteen additional loci constitute potential avenues for future research. Finally, 24 additional independent loci of suggestive significance (*P*_SW_ < *P* < *P*_GW_ and ${{{{\mathcal{C}}}}}_{\ell } > 5$). Several of these have previous evidence of a link to cardiac pathways, for example *RBM20* and genes from the S100A family.

For some loci, a relatively small number of runs produced a latent variable with a genome-wide significant association to the locus: the UPE approach seems crucial for pinpointing this association, as it is likely to be missed in one individual autoencoder run. Also, they are typically missed by shape PCA or handcrafted phenotypes (Table 1). Our approach allows us to detect the milder effect on morphology of common variants near genes whose mutations are known to have highly deleterious effects, either by study of Mendelian diseases in humans or by studies on model organisms. One example of the first is the suggestive association near *FBN1*. It is likely that these variants and the associated unsupervised LV features hold prognostic value; however, this is uncertain at this point, and it should be possible to assess it once UKBB releases more longitudinal data on the same participants studied here.

The SNP rs2245109 is located within the body of the *STRN* gene, on chromosome 2, and is most probably causally related to it. This gene codes for the protein striatin, which is expressed in cardiomyocytes and has been shown to interact with other proteins that affect the mechanism of myocardial function^47^. Mutations in this gene have been shown to lead to DCM in dogs^48^. A recent GWAS on heart failure reported this locus in humans, and our study links it to cardiac morphology. Furthermore, the estimated effect size that we find is notably higher than that for heart failure; this suggests that this latent variable is an endophenotype closer to the underlying biology. This could provide insight to unravel the aetiology of a heterogeneous condition such as heart failure. Furthermore, it makes STRN a promising therapeutic target.

**Extended Data Fig. 5:**
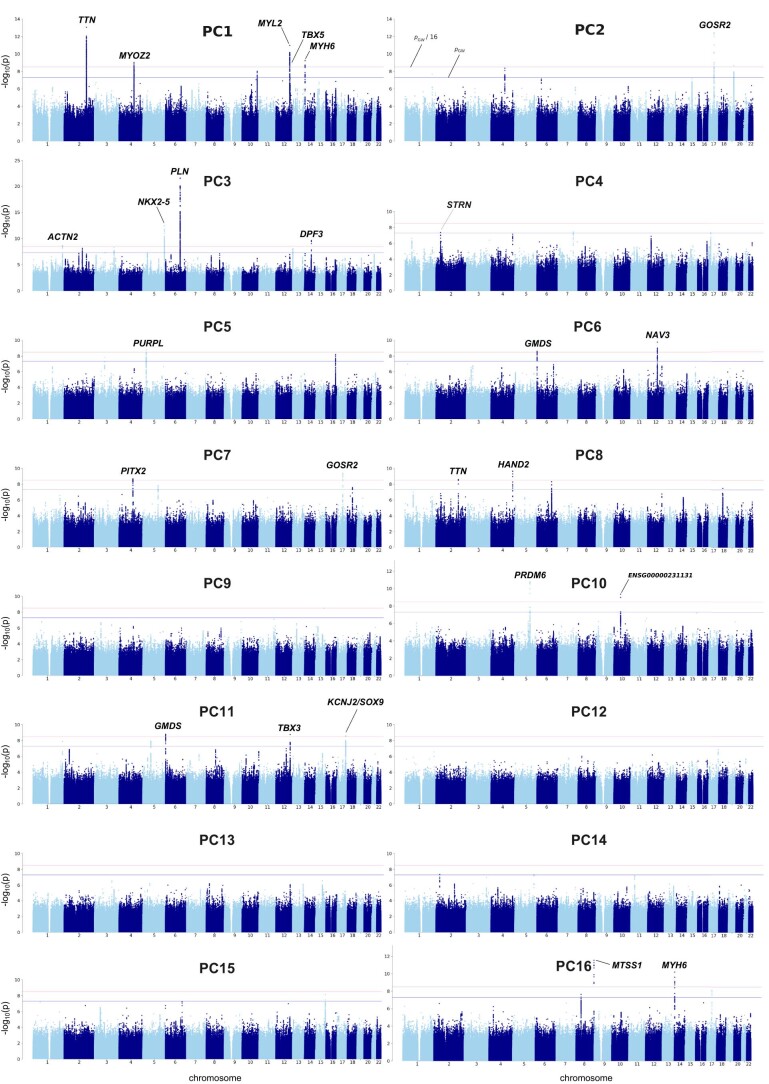
Manhattan plots for GWAS on 16 first shape PCs. Manhattan plot of GWAS for the first 16 shape PCs of the left-ventricular shapes.

As an interesting observation, we note that the phenotypes extracted by UPE and shape PCA show a remarkable oligogenicity, that is, they are controlled by few genes (Extended Data Fig. 5) for shape PCA and Supplementary Figures through for UPE). This is in contrast to what is observed for heterogeneous conditions such as heart failure. For example, heart failure (a single phenotypic score) is linked to 47 loci with genome-wide level of significance^63^. However, a much larger sample size is needed to detect them; indeed, note that this GWAS involves more than 110,000 cases and 1.5 million controls (compared to almost 49,000 participants in our study). Our results confirm (1) the view that endophenotypes are better suited for detecting risk genes for higher-level phenotypes (such as heart failure), due to their higher oligogenicity and stronger link to causal genes (that is, higher effect size) and (2) that the use of unsupervised phenotypes, and in particular the UPE approach, allows one to identify more optimal endophenotypes for each genetic locus, as compared to traditional handcrafted phenotyping approaches, thus boosting discoverability.

In terms of gene discovery, the advantages of an unsupervised phenotyping approach are best conveyed by examining the associated *P* values of the loci found in GWAS performed against traditional handcrafted phenotypes, shown in Fig. 3. For example, when examining the *GOSR2* locus, we found no genome-wide significant association when performing GWAS on traditional LV indices derived from the same meshes; neither have previous studies, except for ref. ^11^ that investigated the trabecular fractal dimension of LV. However, we were able to find it linked to shape PC2, which seems to model LV conicity. Similarly, the UPE approach finds it in 26 (out of 36) runs, where the best latent variable models a similar phenotype (Supplementary Fig. 6). Other examples of novel associations found via shape PCA and UPE are *ACTN2*, *PITX2*, *NAV3* and *PRDM6*.

Likewise, other genes, such as *STRN*, which have previous knowledge of being implicated in cardiac pathways, have not been reported to date in mostly healthy cohorts such as UKBB. It reaches a strong *P* value (*P* = 9.9 × 10^−16^) in our UPE approach, but with shape PCA it only reaches genome-wide significance for PC4, whereas no significant signal is detected for traditional phenotypes. Other examples of highly plausible genes that are found only via UPE are *SHOX2*, *SRL*, *KDM2A*, *NRDG2* and four genes from the *ADAMTS* family.

Some other loci have little evidence to the best of our knowledge, and represent interesting avenues for further research. Examples are the loci near genes *CCDC91*, *FILIP1L* and *CCDC34*, which are of study-wide significance in our approach; however, they have not been reported in previous GWAS on LV phenotypes (that is, all remaining squares are coloured in red shades). Similarly, they are not captured by shape PCA. This highlights the shortcomings of traditional image-derived phenotyping techniques when it comes to the discoverability of relevant genes.

In addition to improved discoverability, the UPE framework enables a more refined understanding of the genetic architecture of cardiac phenotypes, even for genetic loci that were known from previous studies. Most notably, the top SNP in the *TTN* locus was shown to be distinctly related to the size of the LV, while the *PLN* variant (which has been previously found in GWAS of LVEDV) controls a feature that jointly models changes in the size and sphericity of the LV. The *STRN* locus is most strongly associated with a subtle feature that controls mitral orientation and was therefore not discovered in previous studies, which investigated more global phenotypes.

On the basis of our findings, we argue that, in large-scale imaging studies, it is crucial, along with increasing sample size, to count with good techniques to perform deep phenotyping that allow to boost gene discoverability in GWAS.

## Conclusions

In this work, we proposed a framework for LV phenotyping based on unsupervised geometric deep learning techniques in image-derived 3D meshes to discover genetic variations that affect the shape of the LV through GWAS. The proposed methodology is based on finding a latent low-dimensional representation of the CMR-derived LV 3D meshes using CoMAs and then performing GWAS on the learnt latent features. As proposed, this dimensionality reduction method, using Kullback–Leibler regularization, yielded phenotypes with statistically significant genetic associations.

The methodology of ensembling SNP associations across representations obtained through different network metaparameters, followed by the correction in the Bonferroni threshold necessary to control for false discovery rate, has proven effective in identifying novel associations of mesh-derived phenotypes with genetic loci. In addition to previously identified loci, namely *TTN*, *PLN*, *GOSR2* and *ATXN2*, we report 40 additional genetic loci that have not been discovered in recent GWAS of LV phenotypes. Moreover, we report 24 independent associations that do not exceed our corrected Bonferroni threshold; however, their association remains suggestive by virtue of exceeding the usual genome-wide significance threshold of *P*_GW_ = 5 × 10^−8^ in more than five unsupervised phenotypes, obtained from independently trained autoencoder networks. Some of the last genes, such as *S100A1*, *LMCD1*, *RBM20* and *FBN1*, have been previously linked to cardiac pathways.

We argue that the proposed assembly approach is not only useful for discovering novel associations but also enables a deeper understanding of the effect of previously known genes: in fact, the effect of the latent variables with the strongest associations *P* values for each locus can be used as suggestive evidence of the role of that locus in LV shape. For example, we found that the *TTN* and *PLN* variants, which had been previously found to correlate with LV volume, actually have a distinct effect on the shape of the LV. Whereas the *TTN* variant shows in fact a clear effect on LV size, the *PLN* variant is linked to a more complex phenotype that involves a concomitant change in LV volume and sphericity.

More generally, these results validate our methodology to extract knowledge about the genetics driving the morphology of organs, leveraging databases that provide linked genetic and imaging data, such as the UKBB. This methodology can be used seamlessly to study surface meshes of other organs, such as the brain or the skull^64,65^. Additionally, the algorithm proposed here can be extended to process 3D cardiac meshes throughout the cardiac cycle to capture anatomy and quantitative features related to contraction and relaxation patterns. Future studies will explore these directions.

## Methods

The proposed method is outlined in Fig. 1. It starts with extracting 3D meshes representing LV from CMR images using an automatic segmentation method^12^. We then train several models with different metaparameters (network architecture, random seeds controlling weight initialization and dataset partitioning, and relative weight of the variational loss) to learn low-dimensional representations of the 3D meshes that capture anatomical variations using an encoder–decoder model. All meshes are then projected to this latent space to derive a few shape descriptors (or latent variables) for each mesh. To take advantage of the variability induced in the representation obtained by the metaparameters, we pooled the different latent vectors together to obtain a richer representation. The features that make up this pooled representation are finally used in GWAS to discover genetic variants associated with shape patterns.

### Description of the data

The proposed framework can discover novel associations between genetic variations and morphological changes in anatomical structures. We present its potential in the context of cardiac images acquired within the UKBB project (data accession number 11350). The UKBB is a prospective cohort study that between 2006 and 2010 recruited around half a million volunteers in the United Kingdom, aged 40 to 69 years at the time of recruitment^5^. The project collected vast phenotypic information about its participants and linked them to their electronic health records. The collected data includes, among others, genetic data from SNP microarrays for all the individuals and also CMR data for a subset of them (which comprises more than 50,000 individuals at the moment of this writing, but is planned to reach 100,000). These datasets are described in refs. ^66^ and ^67^, respectively.

#### CMR data

The CMR imaging protocol used to obtain the raw imaging data is described in ref. ^67^. We used an automatic segmentation method^12^ to segment the LV in the CMR images. This method generates a set of registered 3D meshes: that is, meshes with the same number of vertices with consistent identical connectivity between them. There is one mesh per participant and per time point. In this work, we only use the LV mesh at end diastole. The LV mesh for the participant *i*, *i* = 1, …, *N*, can then be represented as pairs (**S**_*i*_, *A*), where ${{{{S}}}}_{i}=\left[\,{x}_{i1}\,{y}_{i1}\,{z}_{i1}\,| \,\ldots \,| \,{x}_{iM}\,{y}_{iM}\,{z}_{iM}\,\right]\in {{\mathbb{R}}}^{M\times 3}$ is the shape and *A* is the mesh adjacency matrix. The adjacency matrix is such that *A*_*jk*_ = 1 if and only if there is an edge between vertices *j* and *k* and *A*_*jk*_ = 0 otherwise. The cardiac meshes also have the property of being triangular and closed, so *A*_*jk*_ = *A*_*kl*_ = 1 ⇒ *A*_*jl*_ = 1 for all vertices *j*, *k* and *l*.

#### Genotype data

SNP microarray data are available for all individuals in the UKBB cohort. This microarray covers 801,526, genetic variants that include SNPs and short insertions and deletions. The SNP microarrays used in UKBB have been described in ref. ^66^. An augmented set of more than 90 million variants was imputed from these genotyped markers. GWAS was performed on the latter dataset, particularly on autosomes (chromosomes 1 to 22). The usual quality control steps on the genetic data were performed. This included filtering out rare variants using a threshold for MAF of 1% (within the subcohort of 48,651 participants), a Hardy–Weinberg equilibrium value *P* < 10^−5^ and a low imputation information score (less than 0.3). This results in a set of 9,472,708 genetic variants.

### Unsupervised representation learning for genetic discovery

Given the set of meshes representing the anatomical structure of interest (LV meshes), the pose-sensitive parameters (translation and rotation) were removed using generalized Procrustes analysis. Here we propose to learn a reduced set of features that best describe cardiac shape using CoMA. We will compare the proposed approach with the well-known PCA method. While in PCA only vectorized 3D point clouds **s**_*i*_ will be provided as input (therefore ignoring the data structure and topology), CoMAs leverage topological information about the connectivity between the vertices for learning more powerful nonlinear representations. However, both approaches can be thought of as particular cases of the encoder–decoder paradigm.

In such a model, there is a pair of encoding and decoding functions, ${E}_{\theta }:{{\mathbb{R}}}^{3M}\to {{\mathbb{R}}}^{{n}_{z}}$ and ${D}_{\phi }:{{\mathbb{R}}}^{{n}_{z}}\to {{\mathbb{R}}}^{3M}$ that are parameterized by a set of learnable coefficients *θ* and *ϕ*, respectively. ${n}_{z}\in {\mathbb{N}}$ is the size of the latent space, and is usually chosen so that *n*_*z*_ ≪ *M* (hence the reduction in dimension).

Optimal parameters θ^*^ and ϕ^*^ for reconstruction can be estimated by making the composite function *D*_*ϕ*_∘*E*_*θ*_ as close to the identity function *I* as possible over the training set ${{\mathbb{S}}}_{{{{\rm{train}}}}}\subset {\mathbb{S}}$, using some reasonable measure of reconstruction error *L*_rec_ (examples of which are the norm *L*_1_ norm, the norm *L*_2_ or the mean squared error) along with a regularization term Ω, which will account for additional constraints we want to impose on the model. We want to minimize the following function with respect to *ϕ* and *θ*:

1 $$L({{\mathbb{S}}}_{{{{\rm{train}}}}}| \theta ,\phi )={L}_{{{{\rm{rec}}}}}({{\mathbb{S}}}_{{{{\rm{train}}}}}| \theta ,\phi )+\beta {{\Omega }}({{\mathbb{S}}}_{{{{\rm{train}}}}}| \theta ,\phi ).$$

where $\beta \in {\mathbb{{R}_{\ge 0}}}$ is a weighting coefficient for the regularization term. ${{{{\bf{z}}}}}_{i}:= {E}_{{\theta }^{* }}({{{{S}}}}_{i})\in {{\mathbb{R}}}^{{n}_{z}}$ would then be a low-dimensional representation of the shape *S*_*i*_, while ${\hat{{{{S}}}}}_{i}:= \left({D}_{{\phi }^{* }}\circ {E}_{{\theta }^{* }}\right)({{{{S}}}}_{i})$ is the associated reconstructed shape.

#### PCA

PCA is a standard linear technique for reducing the dimensionality^68^. In terms of the encoder–decoder framework detailed above, it can be obtained by requiring *D* and *E* to be linear transformations and using the norm *L*_2_, in addition to imposing an orthogonality constraint on the latent vectors^69^.

The idea is to find a basis of vectors ${{{{\mathcal{B}}}}}_{{n}_{z}}={\{{{{{\bf{v}}}}}_{i}\}}_{i = 1}^{{n}_{z}}\subset {{\mathbb{R}}}^{M}$ for a fixed *n*_*z*_ < *M*, such that the linear subspace generated by ${{{{\mathcal{B}}}}}_{{n}_{z}}$ captures as much variability in the data as possible. It can be shown that this basis corresponds to the *n*_*z*_ eigenvectors of the covariance matrix of the data, *C*, with the largest *n*_*z*_ eigenvalues; that is, if *C* = *U*^*t*^Λ*U* where Λ = diag(*λ*_1_, *λ*_2_, …), that is it is a diagonal matrix composed of the eigenvalues ordered such that ${\lambda }_{1}\ge {\lambda }_{2}\ge \ldots \ge {\lambda }_{{n}_{z}}$ (all of which are necessarily non-negative). This technique can be used to reduce the dimensionality of shapes or, more generally, point clouds where the vertices are in correspondences. We define, for convenience, the vectorized form of the shapes, ${{{{\bf{s}}}}}_{i}=\left({x}_{i1},{y}_{i1},{z}_{i1},\ldots ,{x}_{iM},{y}_{iM},{z}_{iM}\right)\in {{\mathbb{R}}}^{3M}$. We refer to this approach as shape PCA throughout the text. Given a set of 3D shapes ${\mathbb{S}}={\{{{{{\bf{s}}}}}_{i}\}}_{i = 1}^{N}$, we derive the mean shape $\bar{{{{\bf{s}}}}}$ and the shape covariance matrix *C*:

2 $$\bar{{{{\bf{s}}}}}=\frac{1}{N}\mathop{\sum }\limits_{i=1}^{N}{{{\bf{s}}}},$$

3 $${{{{C}}}}=\frac{1}{N-1}\mathop{\sum }\limits_{i=1}^{N}({{{{\bf{s}}}}}_{i}-\bar{{{{\bf{s}}}}}){({{{{\bf{s}}}}}_{i}-\bar{{{{\bf{s}}}}})}^{t}.$$

In this work, we implemented shape PCA by singular value decomposition of the data matrix (composed of the **s**_*i*_ vectors), using the Python scikit-learn package.

#### CoMA

In an autoencoder, both the encoding and decoding functions are feedforward neural networks. Inspired by recent works on unsupervised geometric deep learning^9^ for facial meshes, we propose the construction of a CoMA that uses spectral convolutions^70^ to learn low-dimensional and nonlinear representations of cardiac mesh structures. Here each layer of the encoder and decoder implements convolution operations parameterized by the graph Laplacian, to leverage information about the local context of each vertex. A hierarchical approach is used to learn global features where each layer of the encoder and decoder implements downsampling and upsampling operations, respectively. Since the vertices are not in a rectangular grid, the usual convolution, pooling and unpooling operations defined for such a topology (usually used in image analysis) are inadequate for this task and must be suitably adapted. Several methods have been proposed to do this^8^, which can be mainly classified into two broad groups: spatial or spectral. The approach proposed in this work belongs to the latter category, which relies on expressing the features in the Fourier basis of the graph, as explained below.

##### Spectral convolutions

The Laplace–Beltrami operator ${{{\mathcal{L}}}}$ (or, more simply, the Laplacian) of a graph with adjacency matrix *A* is defined as ${{{\mathcal{L}}}}:= D-A$, where *D* is the degree matrix, that is, a diagonal matrix with *D*_*ii*_ ≔ ∑_*j*_ *A*_*ij*_ being the number of edges that connect to the vertex *i*. The Fourier basis of the graph can be obtained by diagonalizing the Laplace operator, ${{{\mathcal{L}}}}={U}^{\;t}{{\Lambda }}U$. The columns of *U* constitute the Fourier basis and the operation of convolution ⋆ for a graph can be defined as follows:

4 $$x\star y:= U({U}^{\;t}x\odot {U}^{\;t}y),$$

where ⊙ is the element-wise product (also known as the Hadamard product), and *x* and *y*, are arbitrary functions defined on the graph’s vertices. Spectral methods rely on this definition of convolution and differ from one another in the specific filter used. This work will use a parameterization proposed in ref. ^70^. This method is based on the Chebyshev family of polynomials {*T*_*i*_}. The kernel *g*_*ξ*_ is defined as:

5 $${g}_{\xi }({{{\mathcal{L}}}})=\mathop{\sum }\limits_{i=1}^{K}{\xi }_{i}{T}_{i}({{{\mathcal{L}}}}).$$

*K* is the highest degree of polynomials considered (in this work, *K* = 6). Chebyshev polynomials have the advantage of being computable recursively through the relation *T*_*i*_(*x*) = *xT*_*i*−1_(*x*) − *T*_*i*−2_(*x*) and the base cases *T*_1_(*x*) = 1 and *T*_2_(*x*) = *x*. It is also worth mentioning that the filter described by equation (5), despite its spectral formulation, has the characteristic of being local.

##### Autoencoder

The downsampling and upsampling operations used in this study were proposed in ref. ^9^ based on a surface simplification algorithm proposed in ref. ^71^. These operations are defined before training each layer using a single template shape. Here we use the mean shape $\bar{{{{S}}}}$ as a template.

In each encoder layer, the downsampling operation generates a new triangular mesh (with its corresponding new Laplacian) to minimize the quadric error. Upsampling operations are created while downsampling: the coordinates of the decimated vertices with respect to the remaining vertices are stored for each layer.

##### Variational autoencoder

For some runs, a Kullback–Leibler (KL) divergence term was added to encourage the statistical independence of the different components of the latent representation, which is expected to improve its interpretability^72^. We propose that it will also contribute to producing features with higher heritability, that is, suitable candidate phenotypes on which to perform GWAS.

To train a model with such a loss function, the framework of variational autoencoder is used. In this framework, during the training phase the encoder maps the input into a probability distribution instead of a fixed vector. To emphasize this, we will replace the notation *E*_*θ*_(**S**) for the encoder network with *q*_*θ*_(*Z*∣**S**), the conditional probability of the (now random) latent variable *Z* given the shape S, also a random variable that represents the shapes. During training, for the *j*th latent variable (with 1 ≤ *z*_*j*_ ≤ *n*_*z*_) two quantities are learnt, *μ*_*j*_ and *σ*_*j*_, and a realization *z*_*j*_ of the random variable.${Z}_{j} \sim {{{\mathcal{N}}}}(\;{\mu }_{j},{\sigma }_{j}^{\;2})$ is produced and passed through the decoder to generate the output mesh. The aforementioned Kullback–Leibler-divergence term is then used to encourage the variational approximate posterior to be a multivariate Gaussian with a diagonal covariance structure. The regularization term is computed as:

6 $$\begin{array}{ll}{{\Omega }}({{\mathbb{S}}}_{{{{\rm{train}}}}}| \theta ,\phi )&={{\mathbb{E}}}_{{{{\bf{s}}}} \sim {\hat{p}}_{{{{\rm{train}}}}}}\,{D}_{{{{\rm{KL}}}}}\Big({q}_{\theta }({{{{Z}}}}| {{{\bf{S}}}})| | {{{\mathcal{N}}}}({{{{Z}}}};{{{{0}}}},{{\mathbb{1}}}_{{n}_{z}})\Big)\\ &={{\mathbb{E}}}_{{{{\bf{s}}}} \sim {\hat{p}}_{{{{\rm{train}}}}}}\frac{-1}{2{n}_{z}}\mathop{\sum }\limits_{j=1}^{{n}_{z}}\left(\log {\sigma }_{j}^{2}-{\sigma }_{j}^{2}-{\mu }_{j}^{2}+1\right),\end{array}$$

where ${{\mathbb{1}}}_{n}$ is the identity matrix *n* × *n*, *D*_KL_(*p*∣∣*q*) is the Kullback–Leibler divergence between the probability distributions *p* and *q*, and ${\hat{p}}_{{{{\rm{train}}}}}$ is the empirical probability distribution associated with ${{\mathbb{S}}}_{{{{\rm{train}}}}}$. ${D}_{{{{\rm{KL}}}}}(\;p| | q):= \int\,p(x)\ln \frac{p(x)}{q(x)}{\mathrm{d}}p(x)$. The last equality in equation (6) arises from the formula for the Kullback–Leibler divergence between two normal distributions, where the second is also standardized. During testing, the mode of the latent distribution, *μ*(*S*), is the latent representation of the shape *s*. In the following, we will rename the weighting coefficient *β* of equation (1) as *w*_KL_ to make it more memorable.

### GWAS

According to the traditional GWAS scheme, we tested each genetic variant, *X*_*i*_ ∈ {0, 1, 2}, for association with each latent variable *z*_*k*_ through a univariate linear additive model of genetic effects:

7 $${z}_{k}={\beta }_{ik}{X}_{i}+{\epsilon }_{ik}$$

where *ϵ*_*ik*_ is the component not explained by the genotype, assumed to be normally distributed. The null hypothesis tested is that *β*_*ik*_ = 0.

Only unrelated individuals with self-reported British ancestry were included in the study to avoid problems related to population stratification. This produced a sample size of 48,651 individuals. Summary statistics of demographic data from these subsamples can be found in Supplementary Table 1. For the results presented in the main text, no individuals were excluded according to previous diagnoses or parameters of cardiac function derived from images (such as ejection fraction). Before GWAS, the phenotypes (that is, latent variables) were adjusted for a set of covariates: sex, age, height, weight, body mass index, body surface area, systolic and diastolic blood pressure, alcohol consumption, smoking status and the top ten genomic principal components (computed within the British population). Details on how to compute the genomic principal component loadings and the preprocessing of demographic data are provided in the Supplementary Information (section 1). To make this adjustment, a multivariate linear regression was performed on these covariates and then the residues of this regression were rank-inverse normalized. These inverse normalized residues are the phenotypic scores to be tested in the GWAS.

It is worth mentioning that the GWAS is performed on all individuals, including those on which the dimensionality reduction algorithm was trained. This is correct because the algorithm does not optimize association with genetic variants, and therefore a uniform distribution of *P* values under the null distribution can be safely assumed even when including these participants in the sample.

### UPE

Given that the evaluation metric that guides training, that is, the reconstruction error with variational loss, is not necessarily aligned with the final objective of discovering genes that influence the shape of the LV, there is no reason to adopt the single run with the best value for this metric. This approach was followed in our previous work^10^. Indeed, the observation that several loci are detected in only a small subset of runs indicates that following such a procedure would lead to failure to discover some relevant genetic loci. For this reason, here we propose to adopt an ensemble-based approach, in which we pool the different phenotypes together in a redundant yet more expressive representation. On the basis of the observation that different network metaparameters, dataset partitioning and weight initializations yielded latent representations with different genome-wide significant loci, we proposed building an ensemble of phenotypes by concatenating the latent vectors for each run. This composite representation provides a redundant, yet more expressive representation of the LV shape at the end of the diastole. These runs covered a wide range of *w*_KL_, and variations in network architectures, most importantly in the latent dimension *n*_*z*_. Also, for a given combination of metaparameters (including architecture), an optimal learning rate was found and then five different random seeds were used to initialize the network’s weights and to partition the full dataset into training, validation and test sets (each seed constitutes a different run). Details on the architectural parameters are given in Supplementary Table 2.

#### Run selection

From the complete set of runs, we selected 36 training runs that achieved good reconstruction performance: a root mean squared deviation (r.m.s.d.) of less than 1 mm (averaged over participants from the test set). The deviation is taken to be the vertex-wise Euclidean distance, and the mean is taken over the *M* = 5,220 vertices of the LV mesh. In other words, the r.m.s.d. for participant *i* in run *r* is:

8 $${{{{\rm{r.m.s.d.}}}}}_{i,r}=\sqrt{\frac{1}{M}\mathop{\sum }\limits_{j=1}^{M}| | {{{{\bf{x}}}}}_{i,\;j}-{\hat{{{{\bf{x}}}}}}_{i,\;j}^{(r)}| {| }_{2}^{2}},$$

where **x**_*i*,*j*_ denotes the triad of spatial coordinates for vertex *j* in the mesh of the participant *i*, and ${\hat{{{{\bf{x}}}}}}_{i,\;j}^{(r)}$ is the same for the mesh reconstructed in run *r* of the autoencoder. $| | \cdot | {| }_{2}^{2}$ denotes the squared Euclidean norm. The runs were selected based only on mesh reconstruction error and not in the presence or absence of GWAS hits. This allows us to assume a uniform distribution of *P* values over the [0, 1] interval under the null distribution.

#### *P* value threshold correction

These 36 autoencoder runs produced a total of 384 phenotypes (where the latent dimension was eight for some runs and 16 for others). To control for the false discovery rate, this procedure requires correcting the usual genome-wide Bonferroni *P* value threshold, *P*_GW_ = 5 × 10^−8^, since the number of statistical tests that are performed increases with the size of the (pooled) representation. To avoid overcorrecting this threshold, one was dropped at random whenever a pair of latent variables (within the same run or not) had a Spearman correlation coefficient greater than 0.95 in absolute value. This procedure resulted in *K* = 324 phenotypes to be tested in GWAS. The new study-wide threshold *P*_SW_ is then Bonferroni-corrected dividing the standard genome-wide threshold *P*_GW_ by *K*. Thus, the final threshold is defined as ${P}_{{{{\rm{SW}}}}}=\frac{{P}_{{{{\rm{GW}}}}}}{K}=\frac{5\times 1{0}^{-8}}{324}=1.5\times 1{0}^{-10}$. We note that, given the correlation present between the latent variables, this is a conservative threshold.

#### Genome partitioning and GWAS hit counting

Given that for each genomic locus, the lead variant might vary across different phenotypes by virtue of high linkage disequilibrium with close genetic variants, we adopt the following approach for locus counting: the genome is partitioned into 1,703 approximately LD-independent regions, where each is region is nearly 2 megabases (Mb) in length^45^. We compute the number of autoencoder runs in which each region *ℓ* was genome-wide significant, denoting this quantity ${{{{\mathcal{C}}}}}_{\ell }$: for each run *r* and region *ℓ*, we retrieve the minimum value *p*, across the different latent variables ${z}_{k}^{(r)}$ (recall that 1 ≤ *k* ≤ 8 or 1 ≤ *k* ≤ 16, depending on the run *r*) that we call *p*_*ℓ*,*r*_. We then count the number of runs for which *p*_*ℓ*,*r*_ < *p*_GW_: ${{{{\mathcal{C}}}}}_{\ell }=\mathop{\sum }\nolimits_{r = 1}^{R}{{{{{1}}}}}_{{p}_{\ell ,r} < {p}_{{{{\rm{GW}}}}}}$, where 1 denotes the indicator function and *R* = 36. This ${{{{\mathcal{C}}}}}_{\ell }$ is the quantity labelled ‘count’ in Table 1.

### Downstream analysis of GWAS findings

#### Proximity analysis

We used the Ensembl Biomart database to query the positions of genes surrounding the lead SNPs in each region. We computed the distance between the genetic variant and the TSS and transcription end site (considering the information of the strands present in this database).

#### Transcriptome-wide associations studies

We used the S-PrediXcan tool to assess the correlation between imputed gene expression and intron excision occurrences with the extracted phenotypic data. The primary objective of this analysis is to identify potential candidate genes and the underlying mechanisms that may be involved in the observed genetic associations. The S-PrediXcan tool was supplied with summary statistics from GWAS as well as SNP dosage covariance matrices and gene expression (or intron excision) imputation models that were developed using GTEx data (v.8). These imputation models we used were constructed using the MASHR statistical methodology, which leverages on coexpression patterns across tissues to enhance the precision of estimated effect sizes for expression quantitative trait loci (eQTLs).

### Reporting summary

Further information on research design is available in the Nature Portfolio Reporting Summary linked to this article.

### Supplementary information

Supplementary Information Supplementary Figs. 1–13 and Tables 1–6.

Reporting Summary

Supplementary Data The file contains five tabs: (1) gene-level associations from S-PrediXcan using gene expression models, (2) gene-level associations from S-PrediXcan using intron excision models, (3) pleiotropic effects of discovered loci for all traits, (4) pleiotropic effects of discovered loci for cardiac traits and (5) significant associations for handcrafted LV indices and for the 16 shape PCs.

### Source data

Source Data Fig. 3 −log_10_(*P*) for associations of previous studies, and for our own GWAS on handcrafted LV indices, along with the best −log_10_(*P*) from UPE, for the lead variant in each study-wide significant locus.

## Data Availability

Data for performing the GWAS in this work comes in its integrity from the UKBB. The UKBB Accession code for this application was 11350. Individual-level data are protected and therefore need to be downloaded from the UKBB. 3D mesh data have been produced by ourselves via segmentation of the UKBB CMR imaging data. Interested researchers authorized by UKBB can be advised on how to reproduce these mesh data upon request. Publicly available datasets used for GWAS downstream analyses have been queried for this work: the Ensembl Biomart database (www.ensembl.org), the Integrative Epidemiology Unit OpenGWAS Project (gwas.mrcieu.ac.uk) for GWAS summary statistics, g:Profiler (biit.cs.ut.ee/gprofiler) for gene ontology terms and predictdb.org for GTEx-based prediction models and SNP covariance matrices needed to run S-PrediXcan. In all cases, the date of last access was 12 August 2023. For comparison, GWAS summary statistics were downloaded from http://ftp.ebi.ac.uk using the following study accession codes: GCST009393 through GCST009397 for ref. ^2^, GCST010125 through GCST010131 for ref. ^4^, GCST90000287 through GCST90000295 for ref. ^11^ and GCST90162626 for ref. ^63^. Relevant data for this study has been uploaded to Zenodo: network weights for the ensemble of 36 autoencoders^73^ and the GWAS summary statistics for the traditional indices (LVEDV, LVEDSph, LVM and LVMVR) and for the first 16 shape PCs^74^. A web application has been developed on which researchers can access detailed results derived from this work. Instructions on how to connect to this can be found at www.github.com/cistib/CardiacUPE. Source data are provided with this paper.

## Code availability

The code for this work is split into several repositories publicly available on GitHub. All of them are accessible through a main repository: www.github.com/cistib/CardiacUPE (ref. ^75^). The www.github.com/cistib/CardiacCOMA repository, to which the previous points, is included as a Git submodule and contains the code for an implementation of the Chebyshev-based CoMA, using PyTorch and PyTorch Lightning. Hyperparameters and metrics are logged using the MLflow Python API. This repository also contains code to perform shape PCA on the cardiac meshes, using the scikit-learn Python package. Finally, it contains instructions on how to reproduce the software environment necessary to train the network and produce the latent variables that act as quantitative phenotypes in this work. The second submodule, www.github.com/cistib/GWAS_pipeline, contains the code to carry out preprocessing of genetic data for GWAS, GWAS execution, results visualization and downstream analysis. This repository is written in R and Python, and also contains bash scripts invoking standard GNU command-line tools. Additional tools required for this work are: bgenie, qctool, flashpca, plink and S-PrediXcan.
