# Supplementary material for: Unsupervised ensemble-based phenotyping enhances discoverability of genes related to left-ventricular morphology
Source: Nat Mach Intell. 2024 Mar 11;6(3):291–306. doi: 10.1038/s42256-024-00801-1 (PMC10957472; doi:10.1038/s42256-024-00801-1)
Supplement: Supplementary file 1 — Supplementary Figs. 1–13 and Tables 1–6. [file 42256_2024_801_MOESM1_ESM.pdf]

nature machine intelligence

Article

https://doi.org/10.1038/s42256-024-00801-1

# Unsupervised ensemble-based phenotyping enhances discoverability of genes related to left-ventricular morphology

In the format provided by the
authors and unedited

# Supplemental Materials. Unsupervised ensemble-based phenotyping enhances discoverability of genes related to left-ventricular morphology

## Contents


**1 CMR image and mesh processing** **2**

1.1 Segmentation . . . . . . . . . . . . . . . . . . . . . . . . . . . . . . . . . . . . 2

1.2 Traditional indices . . . . . . . . . . . . . . . . . . . . . . . . . . . . . . . . . 2

**2 Genome-wide association studies** **2**

2.1 Genotype pre-processing . . . . . . . . . . . . . . . . . . . . . . . . . . . . . . 2

2.2 Genome partitioning. . . . . . . . . . . . . . . . . . . . . . . . . . . . . . . . . 2

2.3 Covariates . . . . . . . . . . . . . . . . . . . . . . . . . . . . . . . . . . . . . . 2

Demographic and anthropometric variables • Genetic principal components

2.4 GWAS execution . . . . . . . . . . . . . . . . . . . . . . . . . . . . . . . . . . 3

**3 Mesh dimensionality reduction** **3**

3.1 Shape PCA . . . . . . . . . . . . . . . . . . . . . . . . . . . . . . . . . . . . . 3

3.2 Convolutional mesh autoencoder: implementation details . . . . . . . . . . . . . 4

**4 GWAS results for UPE and downstream analysis** **4**

4.1 Suggestive associations . . . . . . . . . . . . . . . . . . . . . . . . . . . . . . . 4

4.2 Phenome-wide association studies. . . . . . . . . . . . . . . . . . . . . . . . . . 4

4.3 Replication study . . . . . . . . . . . . . . . . . . . . . . . . . . . . . . . . . . 4

4.4 Locus-level figures. . . . . . . . . . . . . . . . . . . . . . . . . . . . . . . . . . 6

Manhattan and LocusZoom plots • Morphological interpretation.

# 1 CMR image and mesh processing

## 1.1 Segmentation

Images were processed through a segmentation pipeline based on the deep learning approach in [12]. This network was trained on a dataset of manually segmented images at end-diastole and end-systole (4700 subjects), for which the manual 2D contours were registered to a 3D cardiac atlas encompassing the 4 chambers [73], producing a 3D mesh for each subject and time point. This set of meshes was used to fit a point distribution model (PDM), consisting of 70 principal components. Briefly, the segmentation approach takes as input a stack of short axis (SAX) views and the different longitudinal axis (LAX) slices, and produces the 70 PCA loadings that allow to reconstruct the mesh for that subject.

It is worth mentioning that the cardiac atlas has a very high resolution, with 194541 nodes for whole heart and 52193 for the left ventricle. Since this makes them too large to be stored permanently for the whole population, they were decimated to 10% and subsetted for the LV, producing the final size of $M = 5220$ nodes. To ensure that all the decimated meshes had the same connectivity and their vertices were in correspondence (a requirement of the dimensionality reduction approach), a suitable decimation approach was used, namely the quadric error minimisation approach [71], which is also used in the pooling layers of the mesh autoencoder.

## 1.2 Traditional indices

The volume of the different chambers was calculated by voxelising the meshes using the capabilities offered by the Trimesh Python library. In particular, the volume for LV at end-diastole was computed (LVEDV).

The segmentation approach yields endocardial and epicardial surfaces for the LV, from which the myocardial mass of this chamber was calculated. This quantity is abbreviated LVM. Also, the left-ventricular mass-to-volume ratio (LVMVR) was computed as a the ratio between LVM and LVEDV.

We did not compute indexed versions of the previous phenotypes as does [4], i.e. phenotypes divided by BSA, since we use this as covariate instead.

The sphericity index for the LV at end-diastole (LVEDSph) was estimated as follows: first, the convex hull (CH) of each cardiac mesh was obtained and, from it, its surface area $A_{\text{CH}}$ and volume $V_{\text{CH}}$. The sphericity was obtained as $\text{Sph} = (36\pi V_{\text{CH}})^{2/3}$, which is tantamount to the inverse of the ratio of $A_{\text{CH}}$ and the surface area of a sphere with volume $V_{\text{CH}}$. Since a sphere is the solid with minimal surface area, this number lies between 0 and 1. To obtain the CH and the associated areas and volumes, the submodule `Spatial` from the `SciPy` Python library was used (version 1.9.3).

# 2 Genome-wide association studies

All the code to perform these steps are included in the public GitHub repository www.github.com/rbonazzola/GWAS_pipeline.

## 2.1 Genotype pre-processing

The pre-processing of the imputed genotypes, consisting of filtering for individuals and genetic variants, was performed using `qctool` (v2.0.8). The SNP and subject filtering, as described in the main text, included excluding rare variants (MAF $<$ 1% within the subcohort of 48,651 British subjects), Hardy-Weinberg equilibrium $p$-value less than $10^{-5}$ and low imputation information score (less than 0.3). This resulted in a set of 9,472,708 genetic variants.

## 2.2 Genome partitioning.

In order to perform locus counting (as detailed in the Methods section), a set of nearly LD-independent regions of around 2Mb were utilised to partition the genome [45].

## 2.3 Covariates

In this subsection, we describe the covariates that were used to adjust the phenotypes tested in GWAS.

### *2.3.1 Demographic and anthropometric variables*

As described in the main text, the demographic variables used as covariates were: height, genetic sex, age, BMI, body surface area (BSA), systolic blood pressure (SBP), diastolic blood pressure (DBP). The respective field codes from the UKBB were 50 ("standing height"), 22001 ("genetic sex"), 21003 ("age when attended assessment centre", instance 2 corresponding to the first imaging visit), 21001 ("body mass index"), 4080 ("systolic blood pressure"), 4079 ("diastolic blood pressure"). BSA was estimated as $0.20247 \times (\text{weight}^{0.425}) \times (\text{height}^{0.725})$, where the weight is expressed in kg and height is expressed in meters. SBP and DBP were adjusted for those participants taking blood-pressure-lowering effect from the verbal interview (field 20003); SBP was adjusted by adding 15 mmHg, whereas DBP was adjusted by adding 10 mmHg (following the procedure in [2]). Smoking status was categorised into 'Current', 'Previous' or 'Never', according to field 20116. Regular alcohol use was

defined as a binary variable based on whether the participant reported consumption of alcohol at least three times per week (field 1558).

Summary statistics for the demographic variables of the cohort used in this study are shown in Table S1.

| | |
|---|---|
| Male proportion | 48.15% |
| Age (years) | $64.7 \pm 15.5$ |
| Height in males (cm) | $176.0 \pm 13.3$ |
| Height females (cm) | $162.6 \pm 12.5$ |
| BMI in males (kg/m$^2$) | $27.0 \pm 7.9$ |
| BMI in females (kg/m$^2$) | $26.1 \pm 9.6$ |
| SBP in males (mmHg) | $142.9 \pm 41.0$ |
| SBP in females (mmHg) | $138.0 \pm 43.2$ |
| DBP in males (mmHg) | $79.8 \pm 14.5$ |
| DBP in females (mmHg) | $76.6 \pm 13.7$ |

**Supplementary Table S1.** Summary statistics of the demographic variables of the subsample of 54,121 unrelated British individuals used in this work (for both the discovery and replication phases). Continuous variables are expressed as mean $\pm$ 2 s.d.

#### *2.3.2 Genetic principal components*

To compute the genomic PCA loadings, a similar approach as detailed in the UKBB genotyping QC report guide was used. It is reproduced here for convenience:

- Minor allele frequency $\geq 2.5\%$ and missingness $\leq 1.5\%$. (Checking that HWE holds in a subset of samples with European descent was part of the SNP QC procedures.)
- Pairwise Pearson $r^2 \leq 0.1$ , to exclude SNPs in high linkage disequilibrium. (The $r^2$ coefficient was computed using `plink` and its `indep-pairwise` function with a moving window of size 1000 bp).
- Removed C/G and A/T SNPs to avoid unresolvable strand mismatches.
- Excluded SNPs in several regions with long-range LD [74]. (The list includes the MHC and 22 other regions.)

We computed the PCA loadings specifically on the individuals self-reported as British, to capture population structure only on this subset.

### 2.4 GWAS execution

GWAS were performed using the BGENIE tool (version 1.4), and executions were conducted on the ARC3 and ARC4 HPCs at University of Leeds, using batch mode. The runs were parallelized using the `Son of Grid Engine` (SGE) job scheduler available in the HPC, and Python scripts to create and submit bash jobs to the queue.

## 3 Mesh dimensionality reduction

Different dimensions of the latent space $n_z$ and weights $w_{\mathrm{KL}}$ were studied, with the aim of achieving a compromise between reconstruction error and interpretability of the components.

Figure S1 shows a comparison of the reconstruction error obtained through principal component analysis (PCA) and convolutional mesh autoencoders (CoMA), as a function of the number of the components $n_z$ of the latent space (values of 8 and 16 were used). CoMA and PCA yielded comparable reconstruction errors, with the best CoMA runs outperforming PCA slightly for $n_z = 8$, and PCA outperforming CoMA for $n_z = 16$. No significant difference was found between non-variational and variational CoMA in terms of the reconstruction error.

### 3.1 Shape PCA

A PCA model was fit using 5,000 subjects. The first 16 components were tested in GWAS and the results are shown in Manhattan plots in the Extended Data Figure 5. The remaining components did not show any significant association.

The effect of the first 8 shape PCA modes are shown in Supplementary Figure S2.

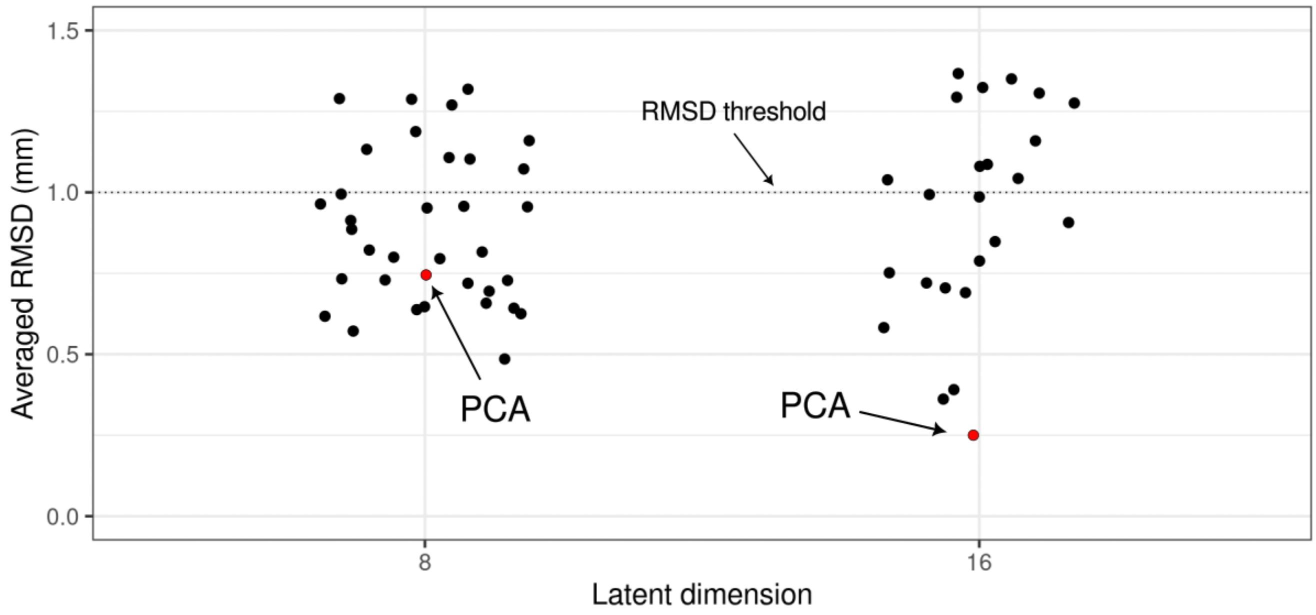


**Supplementary Figure S1.** Averaged reconstruction errors (measured as the RMSD averaged across the test set of 1000 subjects) for different CoMA models (black dots) and for PCA (red dots). A threshold of 1 mm in this metric is used to select the runs.

### 3.2 Convolutional mesh autoencoder: implementation details

For the autoencoder, we ran a grid optimisation scheme for meta-parameter selection, both for the network architecture and the training process. For each number of components $n_z$ and regularisation weight $w_{\mathrm{KL}}$, the execution that presented the minimum mean squared error (MSE) within the validation set was chosen. The autoencoder architecture is detailed in Supplementary Table S2. After each convolutional layer, a ReLU activation function was applied. The number of samples used for training was 5,000, whereas the validation set contained 1,000 individuals. The sample partition, as well as the weight initialisation and the sampling process of the VAE, was controlled by a random seed that was stored along with the trained model for reproducibility; 3 to 5 different random seeds were utilised for each parameter configuration. The Adam optimiser was used to find optimal network parameters, by minimising the KL-regularised MSE reconstruction loss [75]. The learning rate that achieved good performance utilised were in the range $[10^{-4}, 3\times10^{-4}]$.

The network training was performed on a Nvidia DGX A100 workstation located at the University of Leeds. This machine is endowed with Nvidia A100 GPUs. The genetic pipeline was executed on the University of Leeds' high performance computing cluster, ARC. The workload has been parallelised across computing nodes using the Son of the Grid Engine (SGE) queue management system.

## 4 GWAS results for UPE and downstream analysis

### 4.1 Suggestive associations

As described in the main text, we declare a number of loci suggestive based on the following criterion: having a count $\mathscr{C}_\ell \geq 5$ and $p_{\mathrm{SW}} < p_\ell < p_{\mathrm{GW}}$. These loci are given in Table **??**.

### 4.2 Phenome-wide association studies.

To detect pleiotropic effects, we performed a phenome-wide association study (PheWAS) of the lead SNPs from Table 1. For this, we queried the IEU OpenGWAS Project's database. We used the R API implemented in the package `ieugwasr`, version 0.1.5. A subset the GWAS from this database, composed of cardiac traits, for which we used the following regular expression: `"[Hh]eart|[Cc]ardi|ECG|[pP]ressure|Pulse|QRS|PR|QT|P |[Hh]ypertension|[Aa]ort"`. The results are included in the Supplementary Data.

### 4.3 Replication study

We left out a subset of 5,470 UKBB subjects of British ancestry for which the whole pipeline was run identically to the individuals from the discovery set. To make sure the population was identical to that from the discovery set, subjects were

| | Input | Output |
|---|---|---|
| ChebConv | $5220 \times 3$ | $5220 \times C_1$ |
| DS | $5220 \times C_1$ | $2610 \times C_1$ |
| ChebConv | $2610 \times C_1$ | $2610 \times C_2$ |
| DS | $2610 \times C_2$ | $1305 \times C_2$ |
| ChebConv | $1305 \times C_2$ | $1305 \times C_3$ |
| DS | $1305 \times C_3$ | $652 \times C_3$ |
| ChebConv | $652 \times C_3$ | $652 \times C_4$ |
| DS | $652 \times C_4$ | $326 \times C_4$ |
| ChebConv | $326 \times C_4$ | $326 \times C_5$ |
| FC | $326 \times C_5$ | $n_z \times 1$ |

**Supplementary Table S2.** Architecture of the encoder part used for each of the cardiac chambers. The decoder has the same architecture but reading from the bottom upwards and inverting input and output. (ChebConv: Chebyshev convolution, DS: downsampling, FC: fully connected layer.) The architectural hyperparameters were $(C_1, C_2, C_3, C_4, C_5) \in \{(16, 32, 64, 128), (128, 128, 128, 128), (1024, 512, 256, 128)\}$ and $n_z \in \{8, 16\}$.

| chr. | region | candidate gene | count | min. $p$-value | SNP | NEA | EA | EAF (%) | $\lvert\hat{\beta}\rvert \pm \mathrm{se}(\hat{\beta})(\times 10^{-2})$ |
|---|---|---|---|---|---|---|---|---|---|
| 10 | 110317705-112561493 | RBM20 | 21 | $2.8 \times 10^{-10}$ | rs189569984 | C | T | 0.9 | $19.3 \pm 3.4$ |
| 15 | 48136048-50008043 | FBN1 | 21 | $1.7 \times 10^{-10}$ | 15:48690566_TC_T | TC | T | 85.6 | $3.23 \pm 0.96$ |
| 12 | 53039004-54778823 | ATG4D/S1PR5* | 15 | $6.4 \times 10^{-09}$ | rs12810579 | G | C | 1.25 | $19.2 \pm 4.1$ |
| 15 | 84260468-86652905 | ADAMTSL3 | 13 | $3.9 \times 10^{-10}$ | rs2585058 | G | A | 49.5 | $4.02 \pm 0.64$ |
| 20 | 34960446-36909530 | KIAA1755 | 13 | $2.3 \times 10^{-10}$ | rs41282820 | G | A | 1.73 | $10.7 \pm 2.48$ |
| 16 | 60054-1207206 | LMF1* | 11 | $3.8 \times 10^{-10}$ | rs79523980 | T | C | 35.7 | $3.80 \pm 0.69$ |
| 17 | 45876022-47517400 | SKAP1* | 11 | $1.2 \times 10^{-09}$ | rs17697950 | A | G | 24.7 | $2.37 \pm 0.74$ |
| 1 | 59890409-61922365 | NFIA* | 11 | $1.6 \times 10^{-10}$ | rs2474370 | C | T | 67.9 | $2.89 \pm 0.69$ |
| 3 | 170964909-172295731 | FNDC3B | 10 | $7.4 \times 10^{-10}$ | rs17460016 | G | C | 40.1 | $3.34 \pm 0.66$ |
| 17 | 56-1172399 | VPS53* | 9 | $4.0 \times 10^{-09}$ | rs16954854 | G | A | 10.0 | $6.29 \pm 1.07$ |
| 6 | 45406563-47311898 | RUNX2 | 9 | $1.9 \times 10^{-09}$ | 6:45452929_CT_C | CT | C | 24.9 | $3.62 \pm 0.79$ |
| 1 | 153180829-154770403 | S100A1 | 9 | $9.4 \times 10^{-10}$ | rs985242 | C | G | 53.2 | $3.33 \pm 0.65$ |
| 11 | 1213590-3665481 | KCNQ1 | 9 | $4.6 \times 10^{-10}$ | rs569550 | T | G | 38.6 | $3.1 \pm 0.66$ |
| 3 | 137371083-139954597 | NME9* | 8 | $2.9 \times 10^{-10}$ | rs13059110 | G | T | 13.0 | $5.69 \pm 0.96$ |
| 8 | 17387876-17836399 | PDGFRL* | 7 | $5.7 \times 10^{-09}$ | rs2299575 | C | T | 83.1 | $3.88 \pm 0.86$ |
| 1 | 200137649-201589975 | ZNF281 | 7 | $1.2 \times 10^{-09}$ | rs10753873 | A | T | 58.3 | $2.58 \pm 0.65$ |
| 5 | 127344604-129519025 | SLC27A6 | 7 | $1.1 \times 10^{-09}$ | rs1898547 | G | A | 32.1 | $3.79 \pm 0.69$ |
| 4 | 22319347-24135529 | PPARGC1A | 7 | $3.1 \times 10^{-10}$ | rs73243622 | C | T | 25.4 | $4.17 \pm 0.74$ |
| 4 | 174264132-176570716 | HAND2 | 7 | $1.2 \times 10^{-09}$ | rs12502027 | A | G | 36.1 | $2.49 \pm 0.69$ |
| 7 | 116780178-118351581 | WNT2 | 6 | $1.6 \times 10^{-09}$ | rs5004797 | T | C | 18.2 | $4.31 \pm 0.84$ |
| 6 | 1452362-2458936 | GMDS | 6 | $1.6 \times 10^{-09}$ | rs6934958 | T | C | 54.9 | $3.07 \pm 0.65$ |
| 3 | 168580960-170964909 | SAMD7* | 6 | $2.6 \times 10^{-09}$ | rs201527389 | A | AT | 3.1 | $7.83 \pm 1.96$ |
| 3 | 133252173-135456906 | EPHB1 | 6 | $5.3 \times 10^{-09}$ | rs79656429 | A | G | 6.99 | $5.34 \pm 1.28$ |
| 3 | 7083387-8648561 | LMCD1 | 6 | $9.4 \times 10^{-10}$ | rs9814240 | G | A | 42.0 | $2.83 \pm 0.65$ |
| 2 | 54685226-56203345 | EFEMP1 | 6 | $2.0 \times 10^{-10}$ | rs1430197 | G | A | 37.8 | $2.39 \pm 0.67$ |
| 5 | 36433954-38802410 | NIPBL* | 5 | $5.6 \times 10^{-09}$ | rs292178 | C | G | 43.9 | $3.30 \pm 0.65$ |
| 11 | 90966490-92077144 | FAT3 | 5 | $1.2 \times 10^{-08}$ | rs72972727 | T | G | 10.2 | $4.98 \pm 1.06$ |
| 1 | 219590571-221858231 | HLX* | 5 | $4.1 \times 10^{-09}$ | rs115466747 | C | T | 6.04 | $6.16 \pm 1.35$ |
| 17 | 63148128-64800430 | PRKCA | 5 | $1.8 \times 10^{-09}$ | rs569258685 | A | AT | 53.4 | $3.08 \pm 0.66$ |
| 12 | 65559695-67181144 | HMGA2 | 5 | $8.9 \times 10^{-09}$ | rs8756 | C | A | 51.9 | $2.96 \pm 0.64$ |

**Supplementary Table S3.** Summary statistics for the 24 suggestive associations obtained via the UPE framework. The count column presents the number of runs $\mathscr{C}_\ell$ for which locus $\ell$ shows at least one association with $p < p_{\mathrm{GW}} = 5 \times 10^{-8}$ (see details in the Methods section). $p$-values are one-sided and derived from a linear association $t$-statistic (no adjustments were made for multiple comparisons). Note that the total number of runs was 36. Genes with an asterisk were annotated based purely on proximity to the lead variant in that region. Gene names with no asterisk have additional prior evidence of a link to cardiac physiology.

allocated to each set randomly. In particular, the allocation is not associated to the date when the scans were taken, as indeed we found that subjects with more recent scans tend to be older. The results of our validation study are shown in Supplementary

Table S3. For the lead SNP in each region, the best latent variable from UPE (i.e. the one with minimum $p$-value) in the discovery set, was tested within the independent validation set.

## 4.4 Locus-level figures.

Here we present, for a selection of loci, a triad of plots which consists of: 1) the Manhattan plot for the GWAS of the single phenotype (i.e. latent variable) that yielded the strongest $p$-value against that locus, 2) a LocusZoom plot of a 1Mb region centered at the lead SNP for each locus and 3) a sequence of meshes for different ranges of quantiles of the latent variable.

### 4.4.1 Manhattan and LocusZoom plots

Manhattan plots were generated using the R package called `qqman`. LocusZoom plots were generated using the 1.4 version of the tool (which is not being actively maintained anymore). These plots allow to examine the region close to the lead SNPs, see which genes lie in that region; and also, by examining the correlation with nearby SNPs, to visually determine the presence of independent GWAS signals. The LD reference utilised was the European subpopulation of the 1000 Genomes panel (March 2012). Note that some of the genetic variants in the UKB SNP microarray had no available LD information; in that case, they are coloured in grey. Moreover, the plots are annotated with GWAS hits from previous GWAS summary statistics hosted at `www.genome.gov`; however, bear in mind that this list is far from complete.

### 4.4.2 Morphological interpretation.

An interpretation of the impact on LV morphology of the latent variables linked to different loci was achieved by examining the average shape of subjects located at different quantiles. Prior to averaging, the sets of meshes were unscaled, and then scaled back after averaging. The quantile ranges used were: [0, 0.01], [0.095, 0.105], [0.495, 0.505], [0.895, 0.905] and [0.99, 1]. Note that, since there are 48,651 subjects in our database, each quantile range (of 1% in width) encompasses more than 500 subjects. In all cases, we observe a smooth transition in shape from lower to higher values.

An alternative approach that was tested was the following: varying the components of the latent representation one at a time (while keeping the others fixed at the mean value) and generating the associated synthetic shapes by means of the trained decoder. However, note that since $w_{\mathrm{KL}}$ (the parameter that controls the strength of the variational loss) spans a broad range of values in our ensemble of runs, independence of the different latent variables within a run is not guaranteed for all runs (and indeed, it is not observed in most of them). For this reason, it is not possible in general to use the decoder in this way: it is only valid when statistical independence of the latent variables is observed.

Additionally, the Spearman correlation coefficients between these latent variables and the four LV handcrafted phenotypes are provided in Table S3.

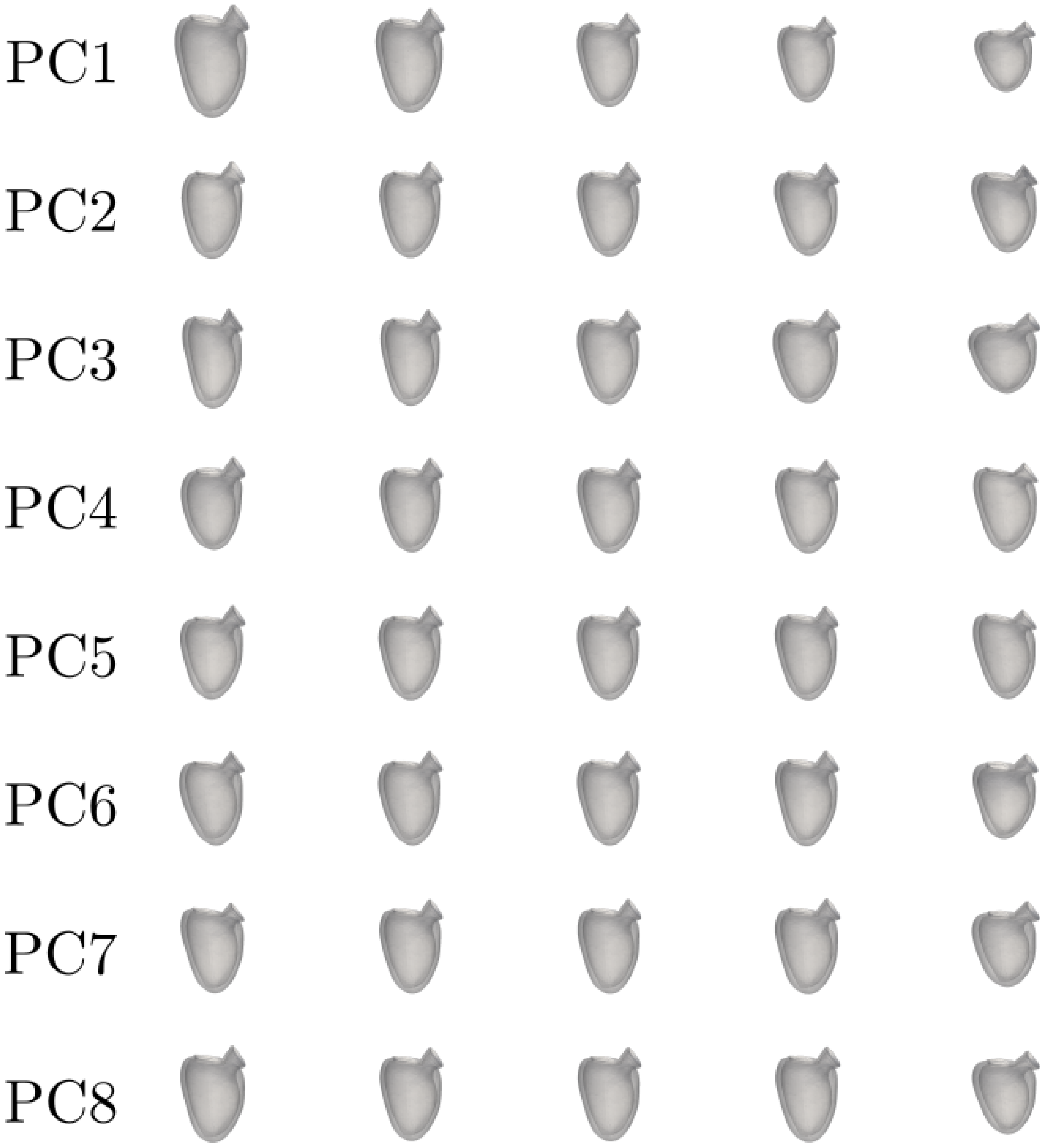


**Supplementary Figure S2.** Effect of the first 8 shape PCs. Each shape corresponds to the average of the following quantiles: [0, 0.01], [0.095, 0.105], [0.495, 0.505], [0.895, 0.905] and [0.99, 1]. As explained in the text, before averaging, the shapes are unscaled, and then scaled back after averaging.

rs11153730, candidate gene PLN (chromosome 6, position 118667522)

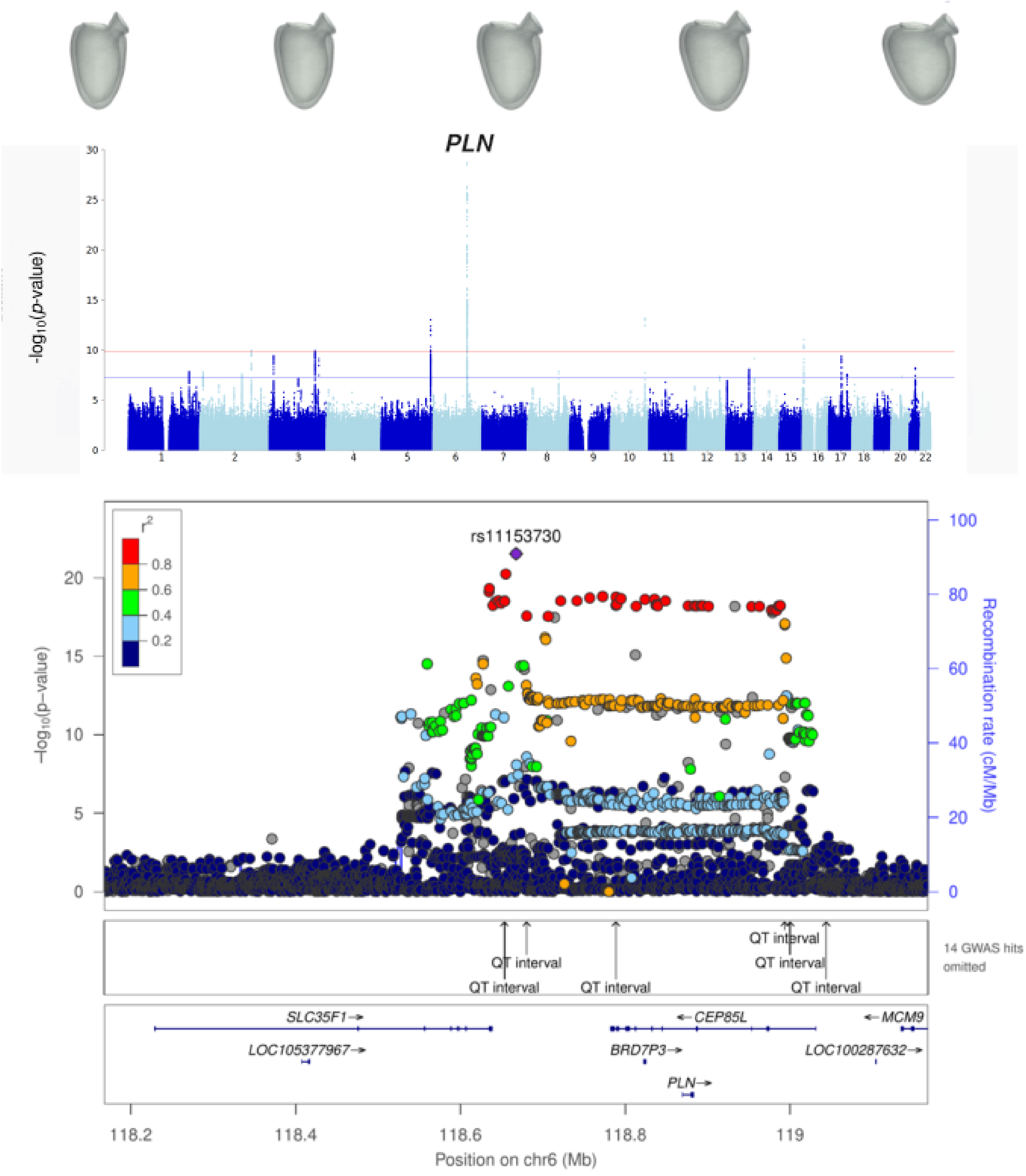


**Supplementary Figure S3.** Triad of plots for locus PLN.

| gene | direction concordance | replication $p$-value | power ($\alpha = 0.05$) |
|---|---|---|---|
| ABRA | ✓ | $3.0 \times 10^{-2}$ | 0.645 |
| ACTN2 | ✓ | $6.8 \times 10^{-2}$ | 0.627 |
| ACVR2A | ✓ | $5.4 \times 10^{-3}$ | 0.622 |
| ADAMTS1 | ✓ | $4.4 \times 10^{-2}$ | 0.569 |
| ADAMTS18 | ✓ | $1.2 \times 10^{-2}$ | 0.665 |
| ADAMTS6 | ✓ | $1.4 \times 10^{-2}$ | 0.614 |
| AFAP1 | ✓ | $2.4 \times 10^{-2}$ | 0.648 |
| BAG3 | ✓ | $3.6 \times 10^{-2}$ | 0.822 |
| CCDC34* | ✓ | $1.4 \times 10^{-1}$ | 0.652 |
| CCDC91* | ✓ | $5.5 \times 10^{-2}$ | 0.719 |
| CDKN1A | ✓ | $5.4 \times 10^{-3}$ | 0.665 |
| DOCK9* | ✓ | $1.6 \times 10^{-1}$ | 0.594 |
| EN1* | ✓ | $5.9 \times 10^{-2}$ | 0.691 |
| FDPS | ✗ | $2.9 \times 10^{-1}$ | 0.665 |
| FGF9 | ✓ | $1.0 \times 10^{-1}$ | 0.699 |
| FILIP1L* | ✓ | $2.6 \times 10^{-3}$ | 0.709 |
| GATA6 | ✓ | $2.5 \times 10^{-2}$ | 0.574 |
| GJA5 | ✓ | $4.8 \times 10^{-2}$ | 0.585 |
| GOSR2 | ✓ | $8.2 \times 10^{-5}$ | 0.892 |
| HEY2 | ✓ | $3.8 \times 10^{-5}$ | 0.617 |
| HSPB7 | ✓ | $5.5 \times 10^{-3}$ | 0.592 |
| IGFBP3 | ✓ | $2.2 \times 10^{-2}$ | 0.623 |
| KCNJ2 | ✓ | $1.3 \times 10^{-1}$ | 0.635 |
| KDM1A | ✓ | $8.1 \times 10^{-6}$ | 0.638 |
| KDM2A | ✓ | $1.7 \times 10^{-1}$ | 0.612 |
| MAF* | ✗ | $3.9 \times 10^{-1}$ | 0.652 |
| MYH6 | ✓ | $4.1 \times 10^{-2}$ | 0.700 |
| MYL2 | ✓ | $7.7 \times 10^{-7}$ | 0.735 |
| MYOZ2 | ✓ | $1.8 \times 10^{-1}$ | 0.697 |
| NAV3 | ✓ | $9.2 \times 10^{-2}$ | 0.638 |
| NDRG2 | ✓ | $3.2 \times 10^{-2}$ | 0.594 |
| NDUFV2 | ✓ | $4.8 \times 10^{-4}$ | 0.634 |
| NKX2-5 | ✓ | $1.1 \times 10^{-1}$ | 0.691 |
| PIGL* | ✓ | $1.4 \times 10^{-3}$ | 0.568 |
| PITX2 | ✓ | $5.5 \times 10^{-2}$ | 0.707 |
| PLN | ✓ | $2.5 \times 10^{-7}$ | 0.960 |
| PRDM16 | ✓ | $4.0 \times 10^{-2}$ | 0.620 |
| PRDM6 | ✓ | $6.0 \times 10^{-2}$ | 0.582 |
| RNF11 | ✓ | $5.5 \times 10^{-2}$ | 0.622 |
| SHOX2 | ✓ | $6.2 \times 10^{-2}$ | 0.740 |
| SOST | ✓ | $2.9 \times 10^{-2}$ | 0.589 |
| SRL | ✓ | $2.7 \times 10^{-2}$ | 0.603 |
| STRN | ✓ | $1.2 \times 10^{-2}$ | 0.766 |
| SYNPO2L | ✓ | $6.0 \times 10^{-1}$ | 0.761 |
| TBX5 | ✓ | $2.2 \times 10^{-1}$ | 0.634 |
| TMEM43 | ✓ | $9.1 \times 10^{-2}$ | 0.601 |
| TTN | ✓ | $2.1 \times 10^{-4}$ | 0.790 |
| WASF3* | ✓ | $4.0 \times 10^{-3}$ | 0.614 |
| WNT16 | ✓ | $2.8 \times 10^{-1}$ | 0.621 |

**Supplementary Table S4.** Replication results for the study-wide significant loci from the discovery phase. The estimated power for each genetic variant is for a level $\alpha = 0.05$.

| gene | direction concordance | replication $p$-value | power ($\alpha = 0.05$) |
|---|---|---|---|
| ADAMTSL3 | ✓ | $9.9 \times 10^{-3}$ | 0.549 |
| ATG4D/S1PR5* | ✓ | $4.1 \times 10^{-1}$ | 0.803 |
| EFEMP1 | ✓ | $5.5 \times 10^{-2}$ | 0.537 |
| EPHB1 | ✓ | $4.0 \times 10^{-1}$ | 0.521 |
| FBN1 | ✓ | $2.7 \times 10^{-2}$ | 0.597 |
| FNDC3B | ✓ | $2.8 \times 10^{-2}$ | 0.524 |
| GMDS | ✓ | $4.2 \times 10^{-2}$ | 0.506 |
| HAND2 | ✓ | $6.1 \times 10^{-3}$ | 0.533 |
| KCNQ1 | ✓ | $1.6 \times 10^{-1}$ | 0.541 |
| KIAA1755 | ✗ | $3.6 \times 10^{-1}$ | 0.577 |
| LMCD1 | ✓ | $8.0 \times 10^{-2}$ | 0.547 |
| LMF1* | ✓ | $1.0 \times 10^{-1}$ | 0.570 |
| NFIA* | ✓ | $1.0 \times 10^{-2}$ | 0.554 |
| NME9* | ✓ | $4.4 \times 10^{-3}$ | 0.574 |
| PDGFRL* | ✓ | $5.9 \times 10^{-1}$ | 0.500 |
| PPARGC1A | ✓ | $3.1 \times 10^{-1}$ | 0.622 |
| RBM20 | ✓ | $4.7 \times 10^{-3}$ | 0.561 |
| RUNX2 | ✓ | $9.3 \times 10^{-6}$ | 0.546 |
| S100A1 | ✗ | $3.3 \times 10^{-1}$ | 0.526 |
| SAMD7* | ✗ | $5.0 \times 10^{-1}$ | 0.568 |
| SKAP1* | ✓ | $3.2 \times 10^{-2}$ | 0.513 |
| SLC27A6 | ✓ | $4.0 \times 10^{-1}$ | 0.530 |
| VPS53* | ✓ | $2.5 \times 10^{-1}$ | 0.510 |
| WNT2 | ✓ | $4.1 \times 10^{-2}$ | 0.523 |
| ZNF281 | ✓ | $8.6 \times 10^{-2}$ | 0.505 |

**Supplementary Table S5.** Replication results for the loci with suggestive significance from the discovery phase. The estimated power for each genetic variant is for a level $\alpha = 0.05$.

| Loci | LVEDV | LVEDSph | LVM | LVMVR |
|---|---|---|---|---|
| NKX2-5 | -0.546 | -0.470 | -0.287 | 0.409 |
| EN1* | 0.165 | 0.678 | 0.161 | 0.020 |
| LMF1* | 0.839 | 0.273 | 0.697 | -0.194 |
| KCNQ1, MYL2 | 0.885 | 0.218 | 0.809 | -0.086 |
| FILIP1L* | -0.815 | -0.121 | -0.749 | 0.077 |
| HEY2 | 0.907 | 0.092 | 0.894 | 0.011 |
| FDPS | 0.197 | -0.526 | 0.251 | 0.090 |
| BAG3, TMEM43 | -0.809 | -0.445 | -0.685 | 0.167 |
| STRN | -0.331 | 0.452 | -0.341 | -0.045 |
| EPHB1 | -0.728 | 0.311 | -0.696 | 0.047 |
| DOCK9* | 0.239 | 0.675 | 0.221 | -0.023 |
| ACTN2 | 0.335 | -0.521 | 0.486 | 0.251 |
| SAMD7* | 0.093 | -0.147 | 0.114 | 0.041 |
| CDKN1A | 0.316 | -0.486 | 0.416 | 0.161 |
| NDUFV2, SKAP1* | 0.436 | 0.351 | 0.480 | 0.112 |
| WASF3* | -0.263 | 0.527 | -0.286 | -0.016 |
| GATA6 | 0.240 | -0.281 | 0.079 | -0.278 |
| KDM2A, CCDC34*, CCDC91* | 0.101 | 0.431 | 0.019 | -0.118 |
| PITX2 | -0.091 | -0.237 | -0.204 | -0.207 |
| MAF* | -0.711 | 0.256 | -0.658 | 0.063 |
| ADAMTS1 | 0.094 | 0.617 | 0.004 | -0.119 |
| KIAA1755 | -0.223 | -0.575 | -0.136 | 0.100 |
| MYH6 | 0.904 | -0.183 | 0.837 | -0.080 |
| PIGL* | -0.499 | -0.122 | -0.605 | -0.198 |
| TTN | 0.910 | -0.187 | 0.855 | -0.067 |
| WNT16 | 0.055 | 0.036 | 0.279 | 0.366 |
| PRDM16 | -0.683 | 0.432 | -0.600 | 0.123 |
| AFAP1 | 0.673 | 0.367 | 0.724 | 0.119 |
| WNT2 | 0.034 | 0.456 | 0.048 | 0.049 |
| HAND2 | 0.250 | -0.306 | 0.246 | -0.009 |
| ZNF281 | 0.755 | -0.286 | 0.819 | 0.133 |
| NME9*, VPS53* | 0.210 | -0.663 | 0.170 | -0.070 |
| ACVR2A | 0.191 | -0.682 | 0.260 | 0.093 |
| FBN1 | -0.381 | -0.264 | -0.282 | 0.139 |
| ADAMTS18 | -0.231 | 0.446 | -0.428 | -0.334 |
| EFEMP1 | 0.518 | 0.334 | 0.398 | -0.146 |
| GOSR2 | -0.748 | 0.064 | -0.771 | -0.070 |
| SRL, PLN, FNDC3B | 0.722 | 0.532 | 0.567 | -0.212 |
| KDM1A | -0.257 | 0.702 | -0.317 | -0.141 |
| RUNX2 | -0.384 | -0.436 | -0.473 | -0.193 |
| PDGFRL* | -0.369 | -0.339 | -0.422 | -0.132 |
| RBM20 | -0.937 | -0.111 | -0.847 | 0.108 |
| ABRA | 0.366 | 0.540 | 0.346 | -0.001 |
| PRDM6 | -0.496 | 0.377 | -0.648 | -0.257 |
| GJA5 | 0.549 | -0.514 | 0.534 | -0.024 |
| ADAMTS6 | -0.249 | -0.549 | -0.311 | -0.134 |
| HSPB7 | -0.761 | -0.148 | -0.578 | 0.281 |
| FGF9, RNF11 | -0.100 | 0.540 | -0.166 | -0.089 |
| LMCD1 | 0.726 | 0.350 | 0.566 | -0.233 |
| SOST | -0.365 | -0.426 | -0.110 | 0.397 |
| KCNJ2, S100A1 | 0.143 | 0.429 | 0.094 | -0.079 |
| MYOZ2 | 0.912 | -0.060 | 0.845 | -0.079 |
| SHOX2 | 0.734 | 0.232 | 0.596 | -0.193 |
| IGFBP3 | 0.886 | -0.035 | 0.880 | 0.034 |
| SLC27A6 | 0.293 | 0.510 | 0.038 | -0.395 |
| NFIA*, PPARGC1A | -0.273 | 0.566 | -0.363 | -0.130 |
| NDRG2 | -0.657 | -0.317 | -0.700 | -0.101 |
| TBX5 | -0.728 | 0.403 | -0.714 | 0.018 |
| ADAMTSL3 | 0.158 | -0.015 | 0.154 | -0.013 |
| ATG4D/S1PR5* | -0.926 | 0.002 | -0.758 | 0.255 |
| NAV3 | -0.341 | 0.670 | -0.380 | -0.062 |
| SYNPO2L | -0.734 | 0.353 | -0.821 | -0.178 |

**Supplementary Table S6.** Spearman correlation between the best latent variable per locus from UPE, and the four LV handcrafted indices. The signs of the correlations were switched so that the effect sizes from Table 1 are always positive, with the choice of effect alleles described in that table.

rs2042995, candidate gene TTN (chromosome 2, position 179558366)

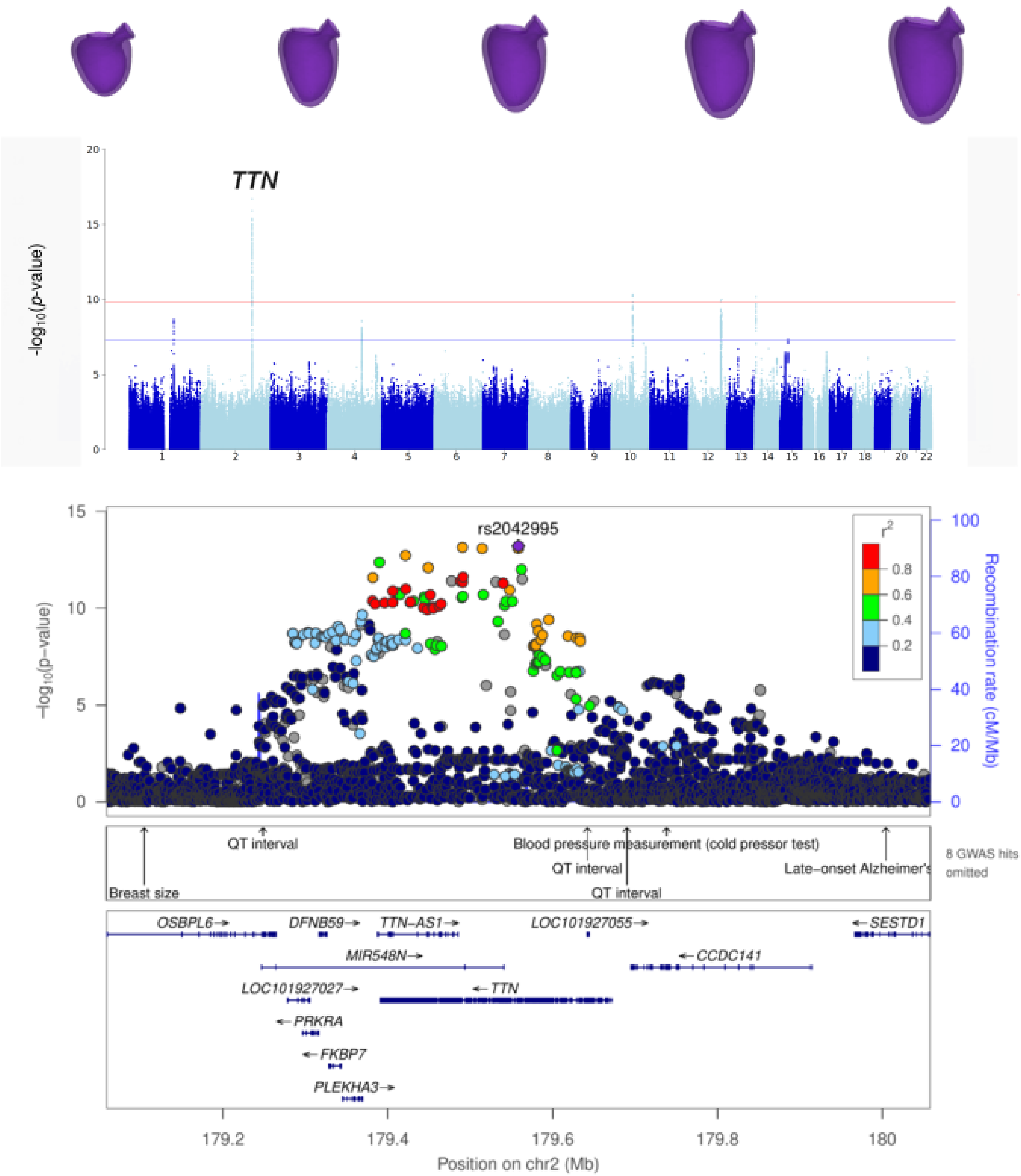


**Supplementary Figure S4.** Triad of plots for locus TTN.

rs4767239, candidate gene TBX5 (chromosome 12, position 114816548)

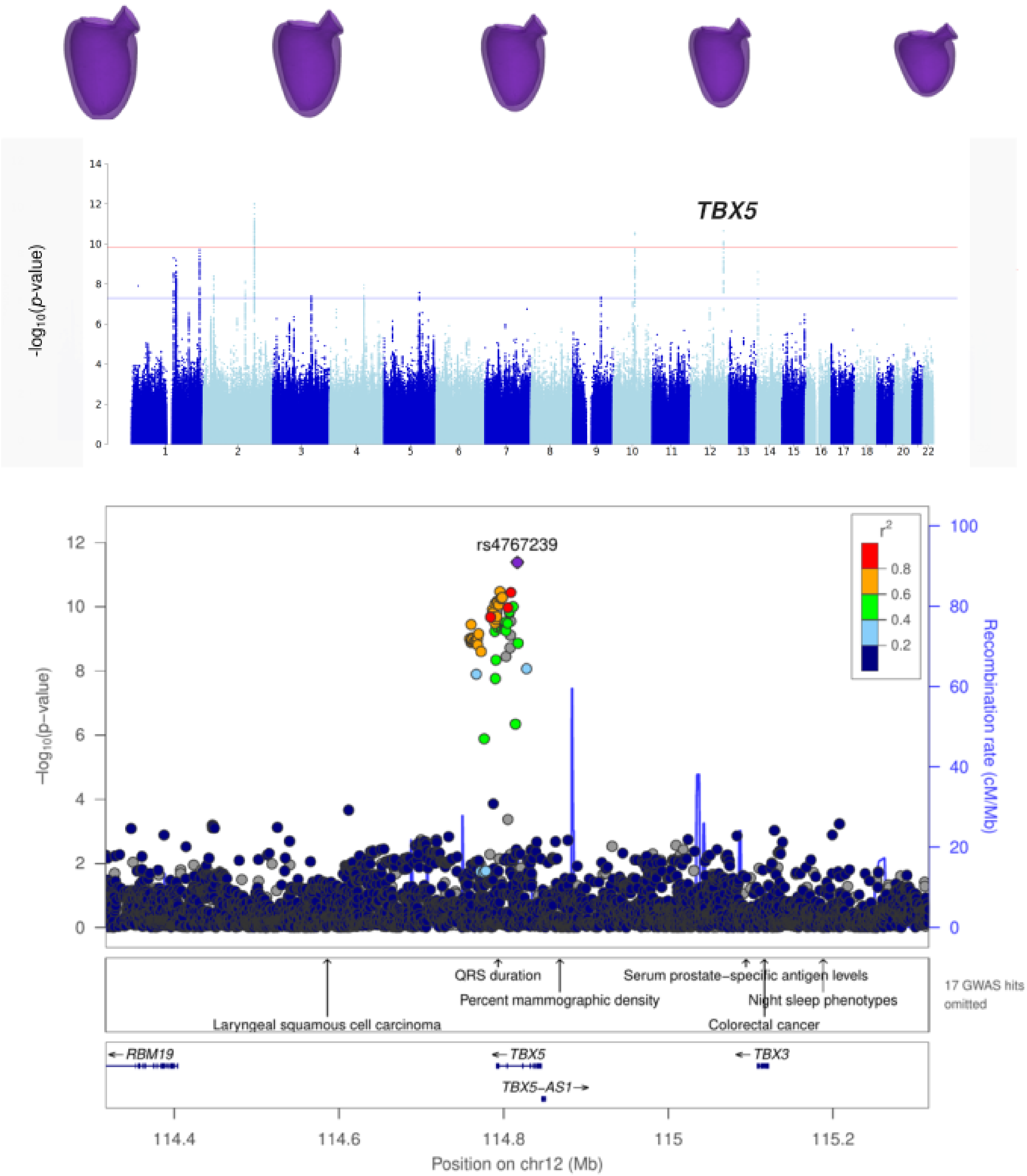


**Supplementary Figure S5.** Triad of plots for locus TBX5.

rs17608766, candidate gene GOSR2 (chromosome 17, position 45013271)

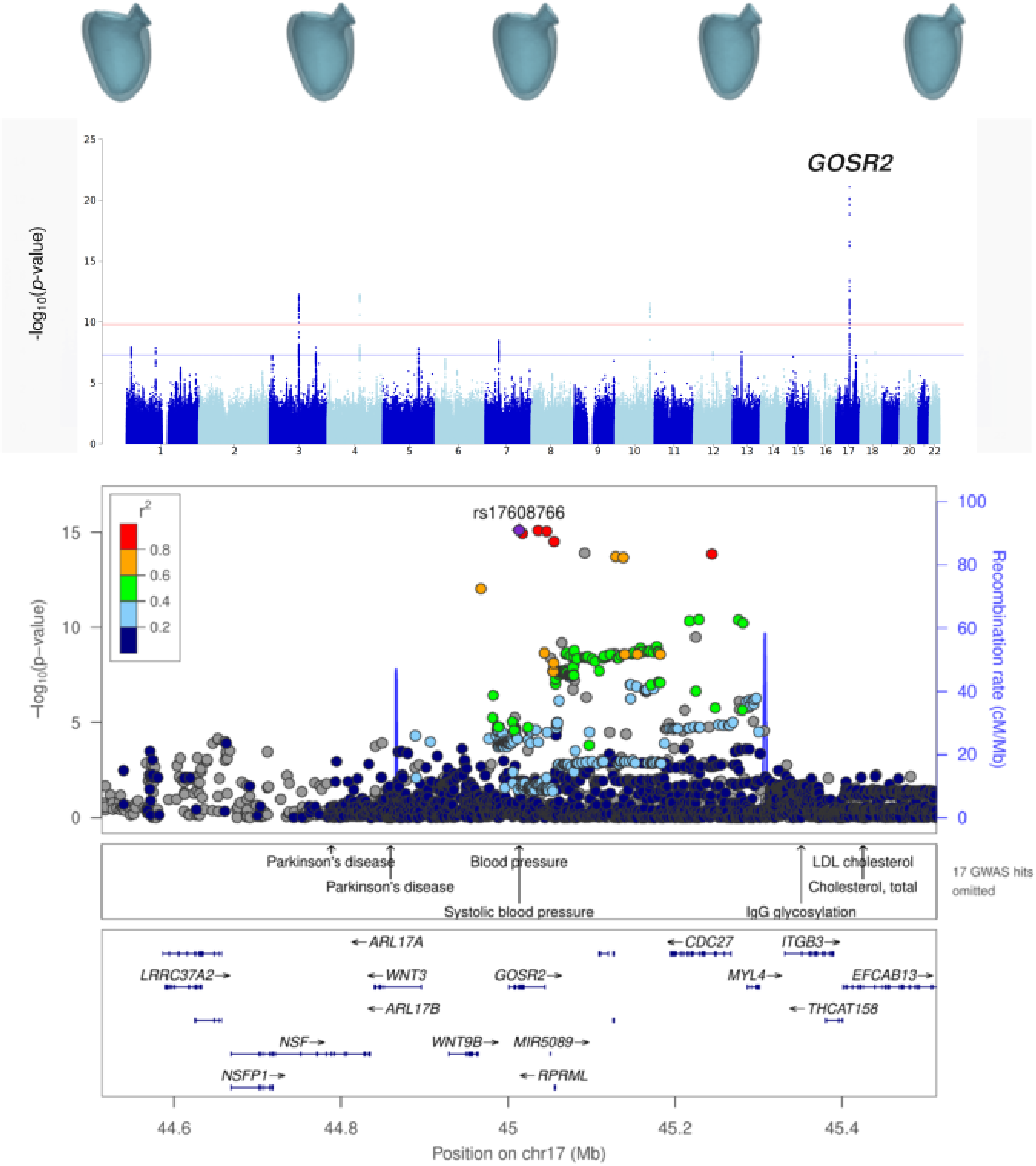


**Supplementary Figure S6.** Triad of plots for locus GOSR2.

rs35564079 (chromosome 5, position 172670611)

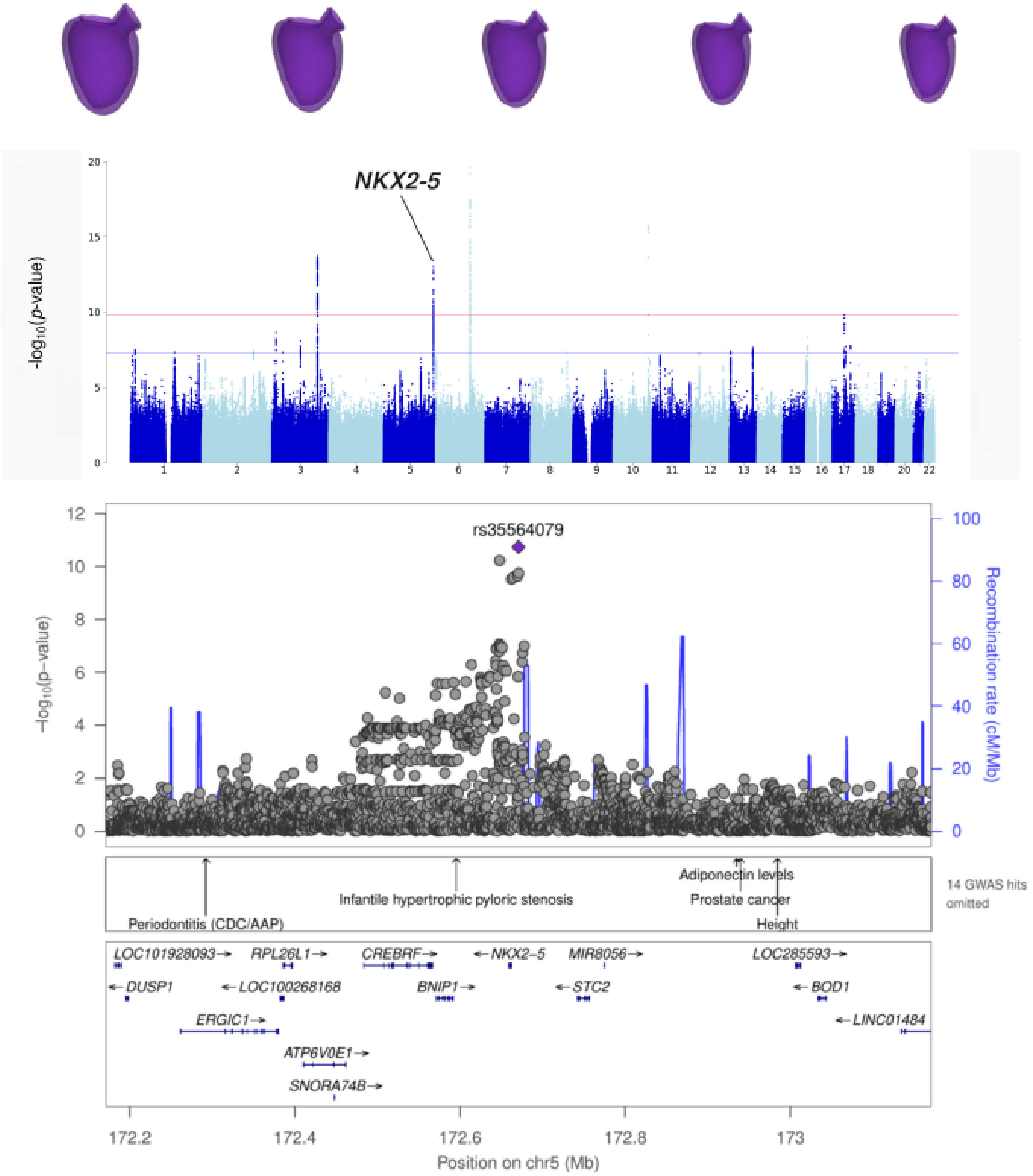


**Supplementary Figure S7.** Triad of plots for locus NKX2.5.

rs189569984, candidate gene RBM20 (chromosome 10, position 112544125)

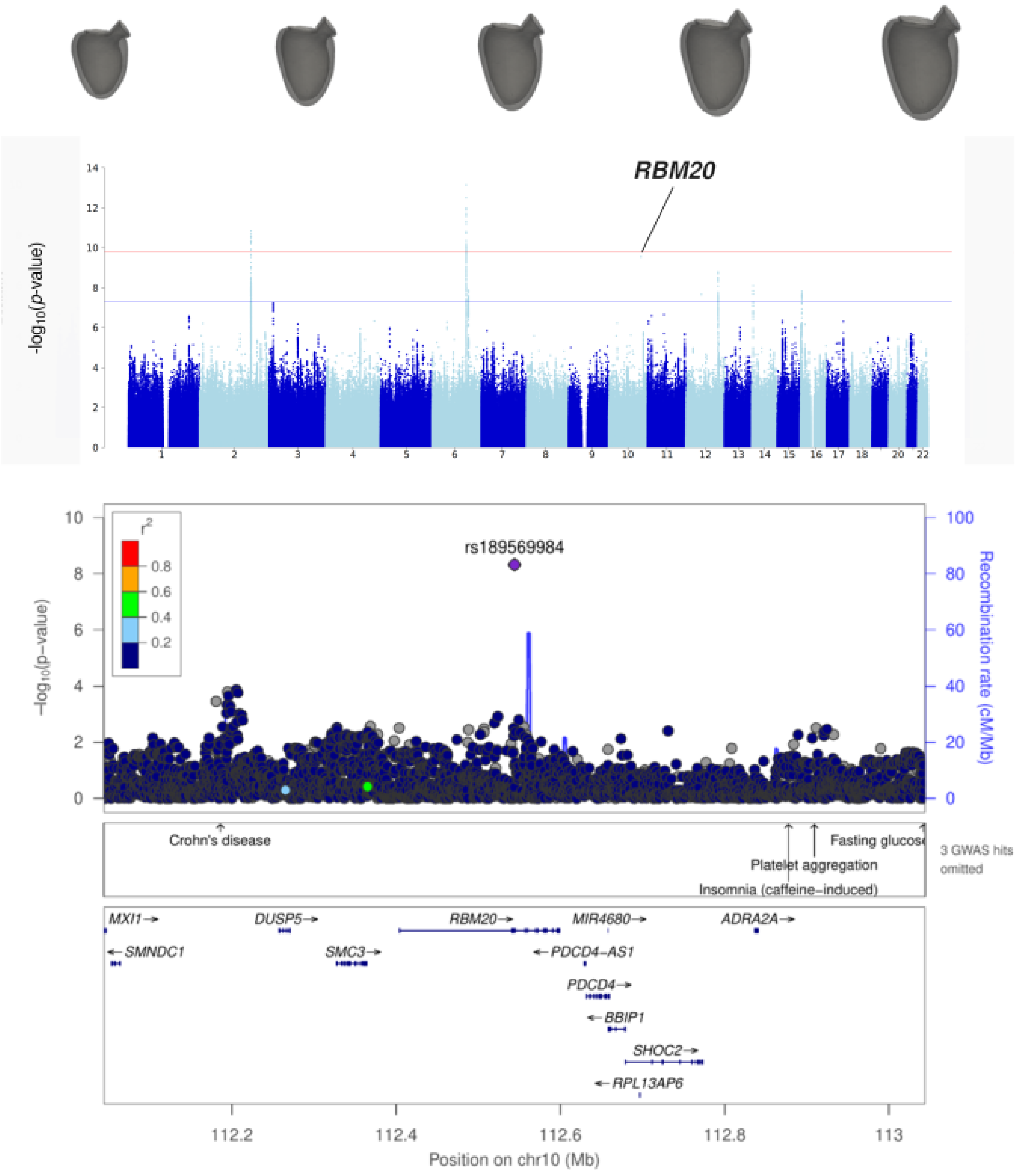


**Supplementary Figure S8.** Triad of plots for locus RBM20.

rs2853621 (chromosome 1, position 236691532)

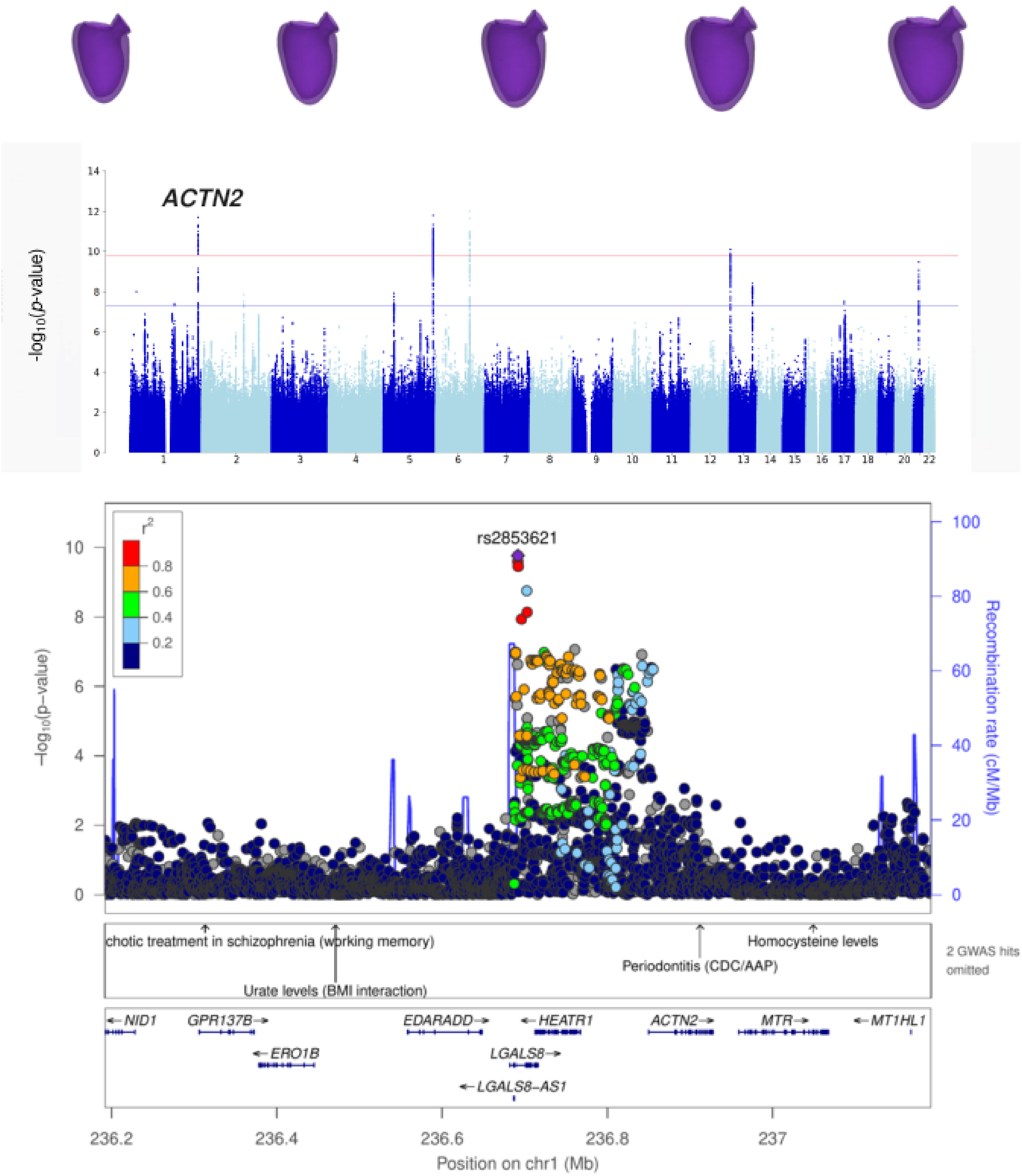


**Supplementary Figure S9.** Triad of plots for locus near gene ACTN2.

rs3741760 (chromosome 12, position 28544464)

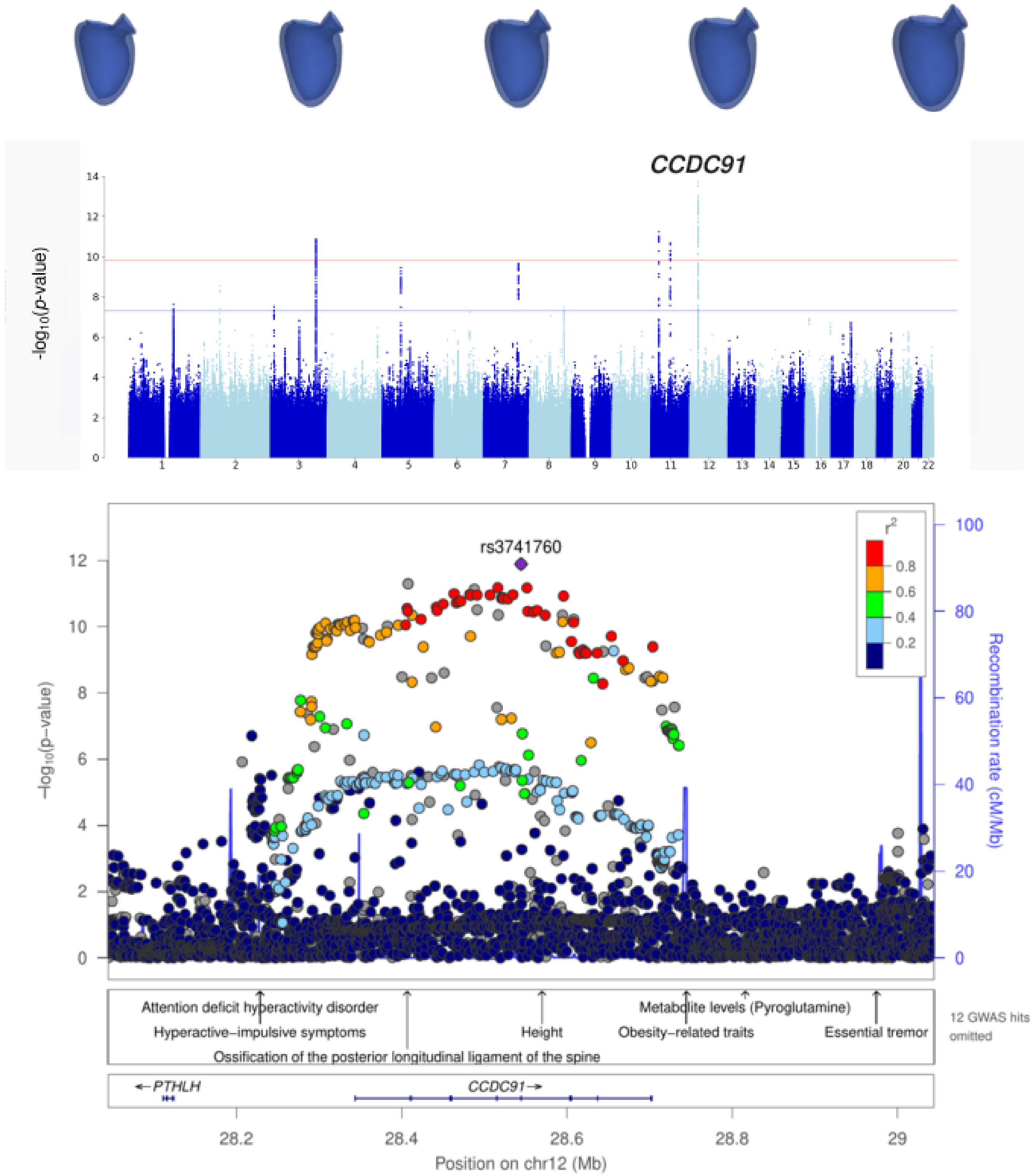


**Supplementary Figure S10.** Triad of plots for locus near gene CCDC91.

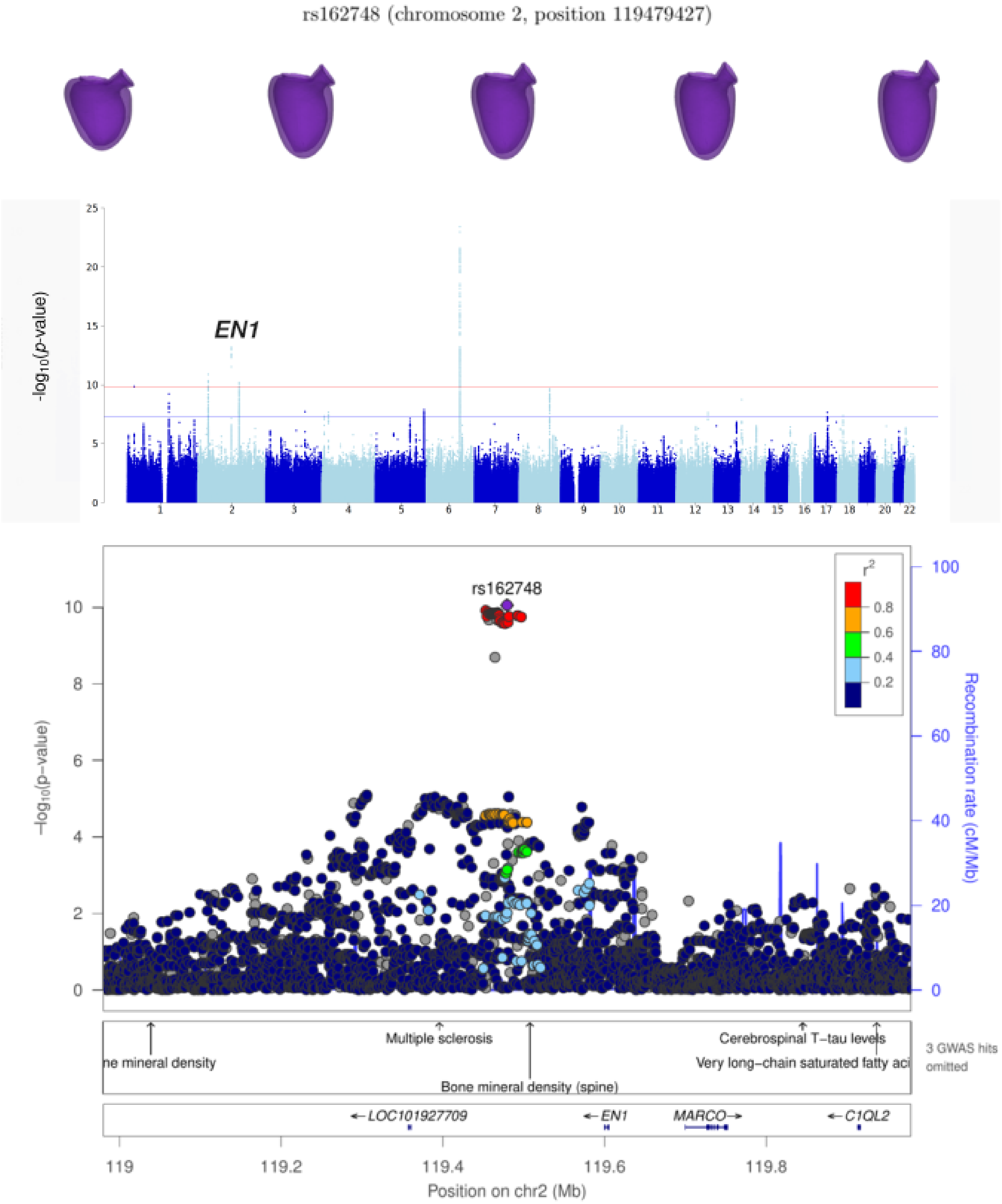


**Supplementary Figure S11.** Triad of plots for locus near gene EN1.

rs375034445, candidate gene BAG3 (chromosome 10, position 121424815)

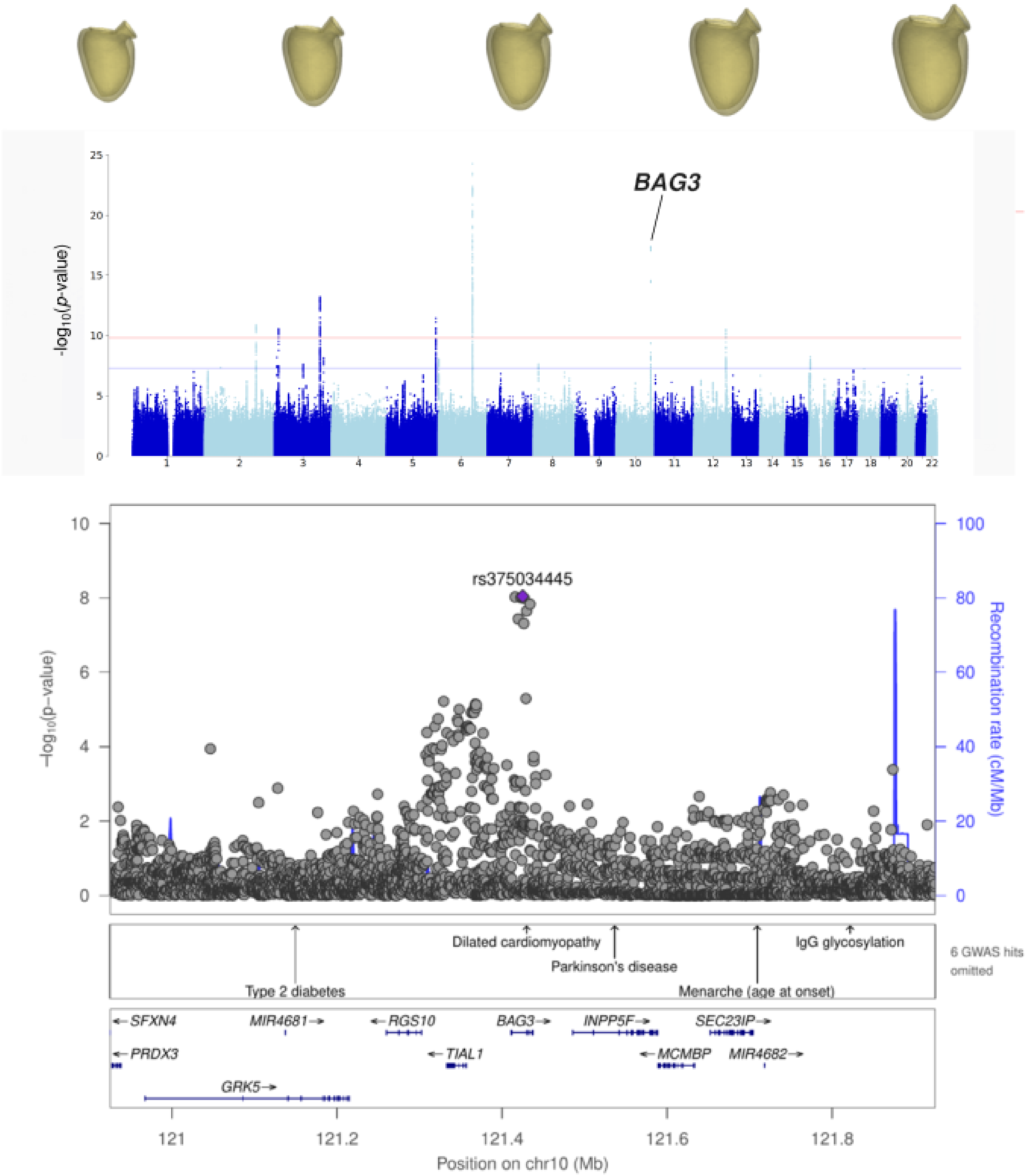


**Supplementary Figure S12.** Triad of plots for locus near gene BAG3.

rs2245109, candidate gene STRN (chromosome 2, position 37086197)

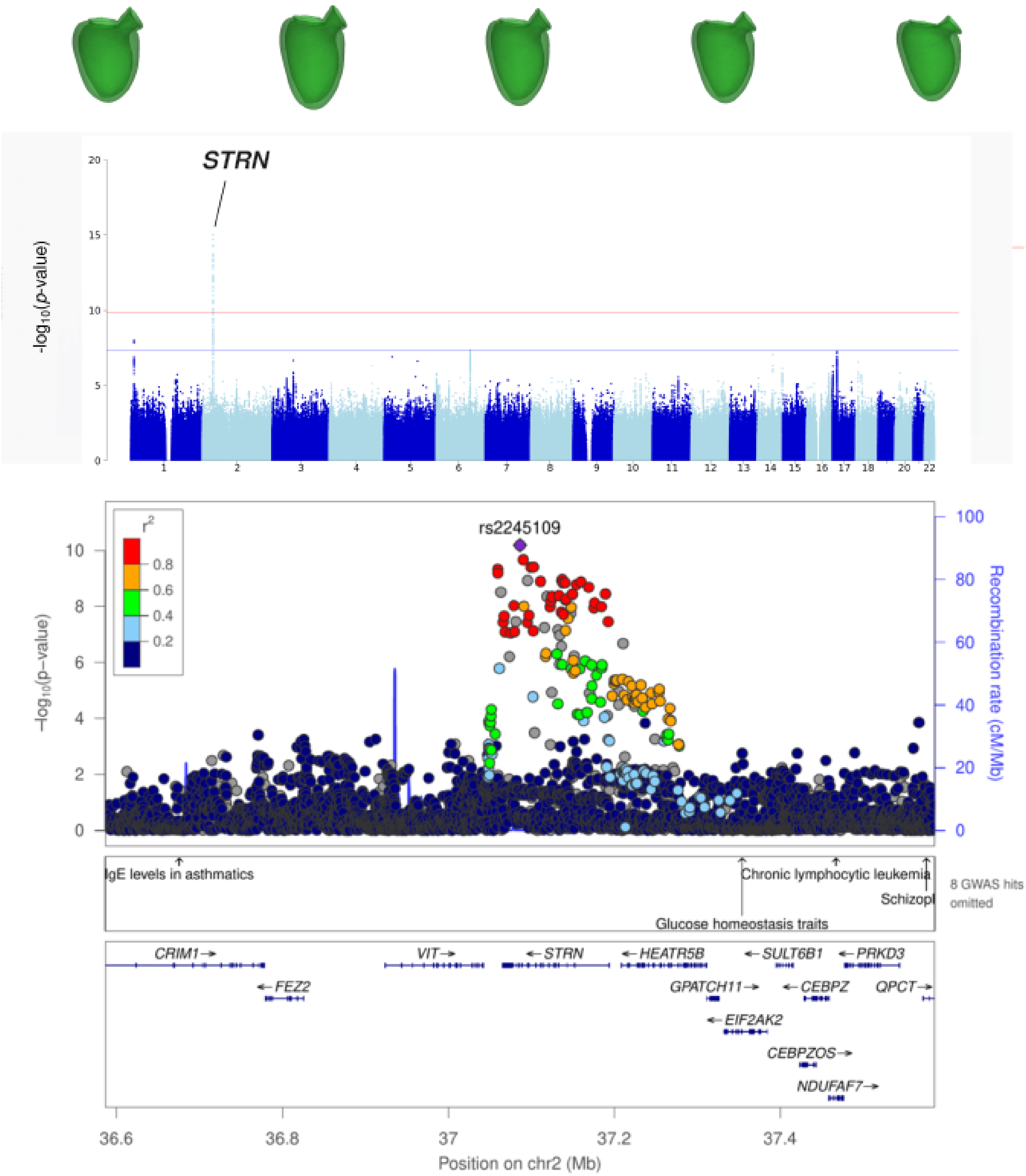


**Supplementary Figure S13.** Triad of plots for locus STRN.
